# Polydatin retards the progression of osteoarthritis by maintaining bone metabolicbalance and inhibiting macrophage polarization

**DOI:** 10.3389/fbioe.2024.1514483

**Published:** 2025-01-07

**Authors:** Qi Sun, Xin-Yu Nan, Hui Wang, Shuo Pan, Gang Ji, Ya-Feng Guo, Ya-Heng Zhao, Gao-Cen Li, Shao-Shi Guo, Lu-Feng Lin, Yu-Jie Jin, Xue Li Zhang, Chang-Cheng Liu, Guo-Bin Liu

**Affiliations:** ^1^ The First Hospital of Hebei Medical University, Shijiazhuang, Hebei, China; ^2^ Hebei Medical University, Shijiazhuang, Hebei, China

**Keywords:** Polydatin, cartilage, extracellular matrix, NF-κB, osteoarthritis

## Abstract

**Background:**

Polydatin (PD), also known as tiger cane glycoside, is a natural compound extracted from the Japanese knotweed plant, which is often referred to as white resveratrol. It exhibits anti-inflammatory, antioxidant, and anti-apoptotic effects in the treatment of various diseases. However, the potential molecular mechanisms of PD in osteoarthritis have not been clearly elucidated.

**Methods:**

Anterior cruciate ligament transection (ACLT) surgery was performed to establish an osteoarthritis animal model. Female mice at the age of 12 weeks were intraperitoneally injected with different concentrations of PD (20 and 40 mg/kg). *In vitro* models were established by isolating mouse articular chondrocytes, which were subsequently treated with lipopolysaccharide or IL-1β for 24 h for subsequent experiments. In addition, different concentrations of PD were administered for 12 h. Morphological changes were observed by toluidine blue staining, joint bone metabolism changes were observed by tartrate-resistant acid phosphatase staining, immunohistochemistry was used to observe the expression levels of inflammatory factors and extracellular matrix. MicroCT analysis was conducted to assess changes in the microstructure of subchondral bone trabeculae, and Western blot was performed to measure the expression of nuclear factor kappa-light-chain-enhancer of activated B cells (NF-κB) signaling pathway and markers of M1 polarization in macrophages.

**Results:**

PD significantly delays the progression of osteoarthritis induced by ACLT, effectively inhibits IL-1β-induced joint inflammation, bone metabolic remodeling and extracellular matrix degradation. In addition, paeoniflorin markedly suppresses the transmission of the NF-κB signaling pathway and reverses M1 polarization in macrophages induced by IL-1β.

**Conclusion:**

Taken together, PD might be a potential therapeutic agent for the prevention and treatment of osteoarthritis.

## 1 Introduction

Osteoarthritis (OA) is one of the most common chronic metabolic joint diseases worldwide, characterized by joint pain, morning stiffness, and loss of joint function. It affects more than 30% of individuals over the age of 65, with a higher prevalence in females than males (Hawker, 2019; Pan et al., 2022). Osteoarthritis often leads to disability, significantly diminishing patients’ quality of life and increasing the economic and healthcare burden on their families and society. In general, the primary pathological features of Osteoarthritis include synovial inflammation, cartilage degeneration, and subchondral bone remodeling (Yue and Berman, 2022). Established risk factors for osteoarthritis encompass a wide range of factors, such as age, hormonal levels, gender, obesity, inflammation, genetics, oxidative stress, trauma, and various other elements (Wang et al., 2023; Driban et al., 2020). Aging and obesity have garnered increasing attention as contributing factors. With the global rise in obesity rates and the advent of an aging population, the number of osteoarthritis patients continues to grow (Yang et al., 2022; Hart et al., 2020). Unfortunately, the pathogenesis of osteoarthritis remains unclear, and effective therapeutic strategies to slow the progression of osteoarthritis are currently lacking. Therefore, gaining a better understanding of the potential molecular mechanisms of osteoarthritis is instrumental in identifying specific therapeutic targets for the treatment of osteoarthritis.

Unlike other tissues, chondrocytes are the sole cell type present in articular cartilage and serve as the primary regulatory units for cartilage metabolism (Piao et al., 2023). Under normal conditions, chondrocytes regulate both synthetic and catabolic processes in cartilage, concurrently secreting a significant amount of extracellular matrix to maintain joint homeostasis (Liu et al., 2022). Aberrations in chondrocyte metabolism are closely associated with phenotypes related to osteoarthritis, including inflammation, apoptosis, necrosis, and senescence, and may indeed be pivotal factors in the initiation and progression of the disease. Inflammation, as reported, plays a significant role in the pathogenesis of osteoarthritis, disrupting the homeostasis of the joint microenvironment, regulating bone metabolism through the NF-κB signaling pathway, and triggering downstream genes, including the conversion of pro-inflammatory cytokines such as IL-1β and IL-18 into mature pro-inflammatory factors, further exacerbating the progression of osteoarthritis (Shi et al., 2022; Sang et al., 2022; Wei et al., 2022). In the context of aerobic metabolism (oxidative phosphorylation) or the oxidation of nicotinamide adenine dinucleotide phosphate (NADPH), reactive oxygen species (ROS) are predominantly produced by the mitochondrial respiratory chain (Napolitano et al., 2021). It has been documented that there is a notable elevation of ROS levels within osteoarthritis tissues, which contributes to the progression of osteoarthritis by perturbing the cellular environment, including the upregulation of inflammatory factor expression (Collins et al., 2018). Moreover, the imbalance in extracellular matrix metabolism is another critical factor in the pathogenesis of osteoarthritis. High expression levels of MMP3, MMP13, and ADAMT4 in articular cartilage of osteoarthritis signify excessive extracellular matrix degradation, thereby accelerating the course of osteoarthritis (Lazarov et al., 2023).

Polydatin (PD), a natural and bioavailable derivative of the potent phytochemical quercetin, is known for its high bioavailability and its beneficial effects, including anti-inflammatory, antioxidant, and anti-apoptotic properties (Karami et al., 2022). Prior research has suggested that PD holds promise in the treatment of inflammation-related conditions such as diabetes, liver damage, cartilage degeneration, and dementia-related diseases (Yousef et al., 2021; Bao et al., 2022; Karami et al., 2022). Studies have demonstrated that PD can potentially address atherosclerosis through its anti-inflammatory, lipid metabolism-regulating, and antioxidative stress-alleviating mechanisms (Huang et al., 2021). Despite ongoing research exploring the potential protective role of resveratrol in osteoarthritis, the precise mechanisms underlying its protective effects in osteoarthritis remain incompletely understood.

In this study, we have demonstrated that PD primarily exerts its therapeutic effects by inhibiting inflammation, apoptosis, abnormal bone metabolism, and extracellular matrix degradation. Additionally, it achieves a significant reduction in Anterior cruciate ligament transection (ACLT) -induced OA by suppressing the activation of the NF-κB signaling pathway, which regulates macrophage polarization. Our findings highlight PD as an effective therapeutic modality for the treatment of osteoarthritis. To delve into the impact of PD on the advancement of OA, this research employs the ACLT method, which is widely accepted, to establish an OA model. Nevertheless, it is essential to recognize that this model has its constraints and may not comprehensively mirror the full range of pathological alterations characteristic of chronic degenerative knee osteoarthritis, particularly those related to hormonal fluctuations and the aging process.

## 2 Materials and methods

### 2.1 Animals

In this study, forty-eight 12-week-old healthy female C57BL/6J mice were purchased from Yi Wei Wo Technology Co., Ltd. (Shijiazhuang, China). The animals were group-housed four per cage under a 12-hour light-dark cycle with access to food and water at a constant temperature of 21°C. All experimental protocols were approved by the Institutional Animal Care and Use Committee. All procedures followed the guidelines of the Animal Care and Use Committee of the Hebei Medical University and admitted by the Animal Ethics Committee of the Hebei Medical University.

### 2.2 Experiment design and operation procedures

Adaptive feeding was conducted for one week, followed by a sham operation (A skin incision was made and then sutured) or anterior cruciate ligament transection (ACLT) on their right knees. All animals were given prophylactic antibiotics (penicillin-G; 40,000 U) for 3 days after the surgery. After ACLT surgery 3 days, the PD group was given intraperitoneal injection of PD 20 and 40 mg/kg/day for 12 weeks. The mice were weighted once a week, and the PD doses was adjusted accordingly. After the treatment, the fresh serum was collected and the right knees were removed.

Half of the samples (n = 6, each group) were used for histological, immunohistochemical analysis. The other half underwent microcomputed tomography (μCT) scanning.

### 2.3 μCT testing

To investigate the alterations in the subchondral bone microarchitecture, fixed joint tissues were scanned with the SkyScan 1176 microcomputed tomography system. We define the trabecular area of subchondral bone under tibial plateau as regions of interest (ROI). We define the trabecular area of subchondral bone under tibial plateau as a ROI. NRecon v1.6 software was used to reconstruct images scanned at 50 kV, 450 μA, and 8 μm. Three-dimensional reconstructed images were obtained by with CTvox v3.0. The CTAn v1.14 and Data Viewer v.1.5 were used to select the ROI.

Transverse images of subchondral bone under tibial plateau were used to measure the microarchitecture after excluding the cortical bone. The joint height index (JHI) is calculated by measuring the height of both sides and the center of the joint space in the mid-sagittal plane for three times, which is a joint height measurement method proposed by our team. An overview of the subchondral bone structure under tibial plateau was obtained by measuring the bone mineral density (BMD), bone volume fraction (BV/TV), trabecular thickness (Tb.Th), trabecular number (Tb.N) and trabecular separation (Tb.Sp) parameters.

### 2.4 Histology and immunohistochemistry examinations

A two-month decalcification in 10% EDTA-2Na was performed after fixation in 10% neutral paraformaldehyde for 48 h. We dehydrated and embedded the paraffin-embedded decalcified samples. The sections were then cut into 6-μm slices for Toluidine Blue (TB) and immunohistochemistry staining. TB was performed using DB0057 per the manufacturers’ directions to assess osteoarthritis-associated degeneration, such as structural disorganization, cartilage injury, and clefts. This analysis was performed by two independent researchers in a blinded manner.

### 2.5 Chondrocyte isolation and treatment

Articular cartilage was separated from the femoral heads of mice, and chondrocytes were separated as previously described. In short, the mice were killed and disinfected in 75% alcohol for 5 min. The entire articular cartilage was separated from the femoral heads and cut into pieces. 0.25% trypsin and 0.2% collagenase II were digested for 40 min and 4 h, respectively. Then, the cells were filtered through a 70 μm cell filter and washed three times with PBS. Subsequently, the collected chondrocytes were cultured in Dulbecco’s Modified Eagle Medium (DMEM) at 37°C, 5% CO_2_. The culture medium was changed every two to three days. The chondrocytes were used for subsequent experiments.

To induce inflammation and cell death by apoptosis, IL-1β (10 ng/mL; Sigma-Aldrich) was added to the culture medium and incubated for 24 h. Then, the cells were treated with different concentrations of PD (20, 40 μM) for 12 h, and the cells were collected for subsequent experiments.

### 2.6 Real-time polymerase chain reaction (RT-PCR) analysis

Samples for RT-PCR were obtained from the chondrocytes. A Gene Amp 7,700 Sequence Detection System (Applied Biosystems, Foster City, CA, United States) and SYBER^®^ Premix Ex Taq™II kit (Takara, Kusatsu, Japan) were used for the RT-PCR. The primers for the selected genes are listed in Table 1. GAPDH was used as an endogenous control. The changes in relative mRNA transcript levels were calculated using the 2 (-Delta C(T)) method as previously described. The experiment was repeated at least three times to ensure accuracy.

**TABLE 1:** Sequences of primers used for RT-PCR.

Gene	Forward primer (5′-3′)	Reverse primers (5′-3′)
GAPDH	GGG​GAG​CCA​AAA​GGG​TCA​TCA​TCT	GAG​GGG​CCA​TCC​ACA​GTC​TTC​T
IL-6	GGC​GGA​TCG​GAT​GTT​GTG​AT	GGA​CCC​CAG​ACA​ATC​GGT​TG
TNF-α	GGA​ACA​CGT​CGT​GGG​ATA​ATG	GGC​AGA​CTT​TGG​ATG​CTT​CTT
iNos	CTC​TTC​GAC​GAC​CCA​GAA​AAC	CAA​GGC​CAT​GAA​GTG​AGG​CTT
Col II	TGT​TTG​CAG​AGC​ACT​ACT​TGA​A	ACC​AGG​GGA​ACC​ACT​CTC​AC
Aggrecan	GAA​GTG​GCG​TCC​AAA​CCA​AC	AGC​TGG​TAA​TTG​CAG​GGG​AC
ADAMT4	CGT​TCC​GCT​CCT​GTA​ACA​CT	TTG​AAG​AGG​TCG​GTT​CGG​TG

### 2.7 Western blot analysis

The chondrocytes were collected and dissolved in a radioimmunoprecipitation analysis buffer using a cocktail of protease inhibitors. The protein lysate was electrophoresed with sodium dodecyl sulfate-polyacrylamide gel and transferred to nitrocellulose membranes. Next, 5% bovine serum albumin was used to block the membranes at 4°C for 2 h. The membranes were subsequently incubated with the following primary antibodies at 4°C overnight: Caspase3 (1:400, Abcam), RIPK1 (1:500, Abcam), RIPK3 (1:500, Abcam), TNF-α (1:200, Abcam) p-IκBα (1:400, GeneTex), IκBα(1:400, GeneTex), p-P65 (1:500, GeneTex), P65 (1:500, GeneTex), p-STAT1 (1:200, Abcam), STAT1 (1:200, Abcam), p-STAT3 (1:400, GeneTex), and STAT3 (1:400, GeneTex). After washing with TBST, the membranes were incubated with HRP-conjugated secondary antibodies at a dilution of 1:2000 at 4°C for 4 h. The proteins were visualized using an enhanced chemiluminescence kit (Merck Millipore, Billerica, MA, United States) and an imaging system (Bio Spectrum 600; UVP, Upland, CA, United States).

### 2.8 ROS detection

The intercellular ROS levels were detected by dichlorofluorescein fluorescence as previously described (Rastogi et al., 2010). Briefly, cells were treated by 5 ng/mL IL-1β for 24 h and then treated with different concentrations of PD (20 and 40 μM) for another 12 h. Subsequently, cells were incubated in 2.5 μM DCF in DMEM at 37°C in the dark for 30 min. The ROS level was detected by flow cytometry. The mean fluorescence intensities for DCF were analyzed by Flow-Jo (BD FACSCalibur; BD Biosciences).

### 2.9 Molecular modelling

The compound structure file (CID: 5281718) of the PD was downloaded from the Pubchem database (https://pubchem.ncbi.nlm.nih.gov/), and the PDB format structure file (PDB ID: 1u3j) of the protein IL37 was downloaded from the PDB database (https://www.rcsb.org/). Pymol-2.1.0 software was used to remove water molecules and small molecular ligands from the target, and AutoDock Tools-1.5.6 was used to hydrogenate and compute charge the protein and save it as pdbqt format. The energy of the compound is minimized using the PyRx built-in tool (OpenBabel). Proteins were used as receptors and compounds as ligands. vina 2.0 inside PyRx software was used to perform molecular docking, calculate binding energy and output result files. Finally, PyMol software was used to visualize the results. The Affinity (kcal/mol) value represents the binding capacity of the two, and the lower the binding capacity, the more stable the binding between the ligand and the receptor, adopt the Discovery Studio 2020 Client (https://discover.3ds.com/discovery-studio-visualizer-download) for visual analysis (The lower the binding energy, the better the binding).

### 2.10 Statistical analysis

The data in this study are presented as the mean ± standard deviation. SPSS 26.0 version was used for all statistical analyses. Normal distribution and homogeneity of variances were evaluated by Shapiro–Wilk and Bartlett’s tests. One-way analysis of variance and Fisher’s protected least significant difference test were adopted for analyzing significant differences. The Kruskal-Wallis test was used to analyze the histological scores.

## 3 Results

### 3.1 Polydatin alleviates damage to ACLT-induced osteoarthritic cartilage

**FIGURE 1:**
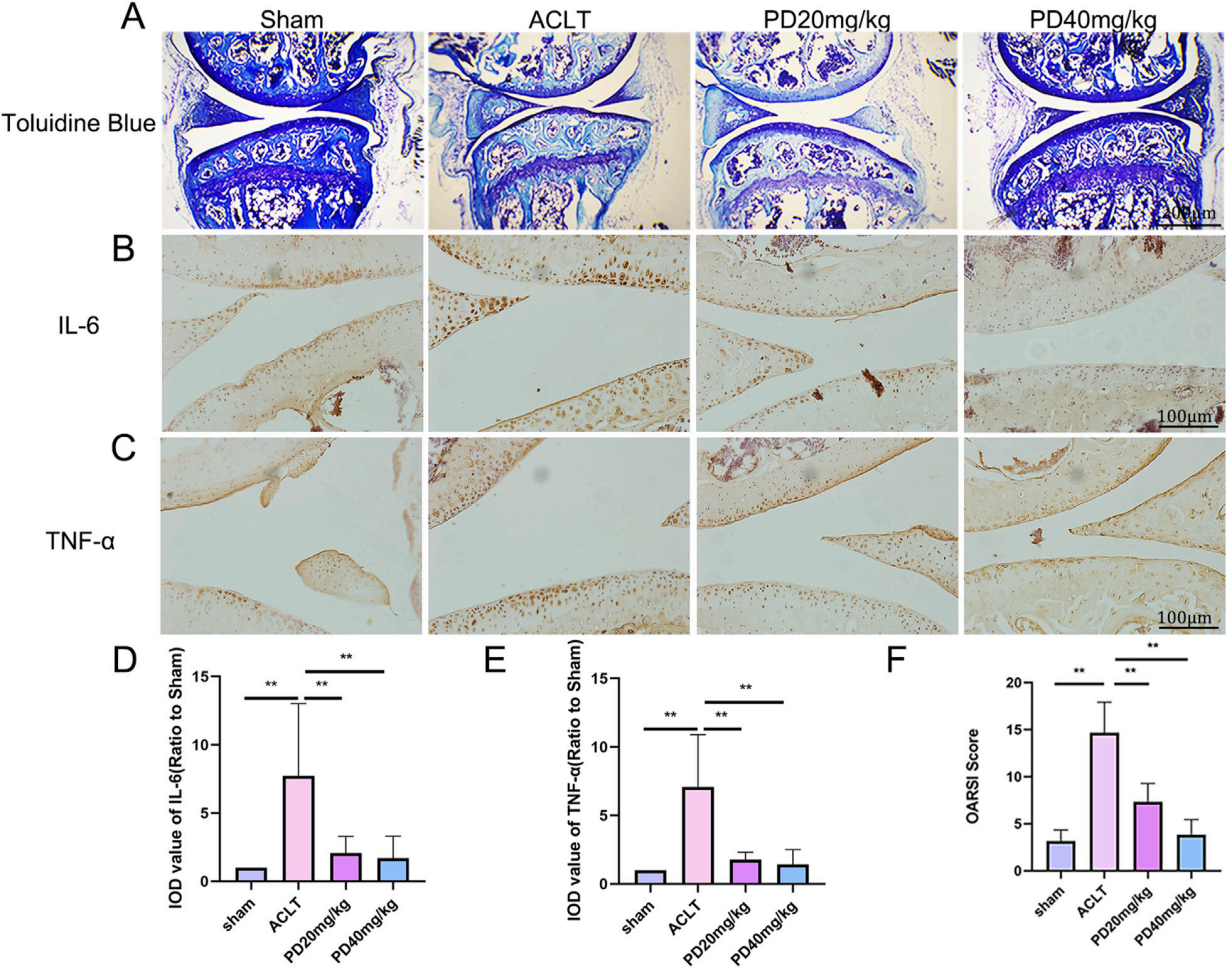
PD attenuated cartilage destruction and inflammatory development in ACLT mice. After ACLT surgery for 3 days, mice were intraperitoneally injected with different concentrations of PD (20 and 40 mg/kg) and maintained for twelve weeks. **(A)** Representative images of Toluidine Blue in knee joints. Immunohistochemistry assay for **(B)** IL-6 and **(C)** TNF-α in the knee joints among all groups. **(D, E)** Immunohistological analysis showed that the expression of IL-6 and TNF-α in the ACLT group was greater than that in the PD groups. **(F)** The quantification of Osteoarthritis Research Society International (OARSI) score in different groups. Note: *P < 0.05, **P < 0.01.

In our study aimed at examining the protective effects of PD on OA, we developed an ACLT-induced OA model and treated OA mice with varying concentrations of PD (20 and 40 mg/kg/day over a 12-week period). Consistent with our expectations, Toluidine Blue staining demonstrated that the ACLT group showed pronounced cartilage degeneration compared to the Sham group, characterized by a marked increase in glycosaminoglycan depletion within the extracellular matrix and more advanced cartilage erosion. Post-PD intervention, however, we observed an enhancement in the smoothness of the cartilage surface, with chondrocytes appearing more numerous, well-filled and orderly arranged. Furthermore, PD was found to notably slow the progression of ACLT-induced OA in a dose-dependent fashion, a finding corroborated by our scoring data presented in Figure 1. In addition, we evaluated the articular cartilage expression levels of inflammatory cytokines IL-6 and TNF-α across all groups. Our results indicated a significant decrease in IL-6 and TNF-α expression in the articular cartilage of PD-treated mice when compared to the ACLT group, as depicted in Figure 1. Collectively, these observations suggest that PD has the potential to suppress the advancement of ACLT-induced OA by modulating the inflammatory response.

### 3.2 Polydatin improves the stability of subchondral bone trabecular microstructure in ACLT-induced osteoarthritis

**FIGURE 2:**
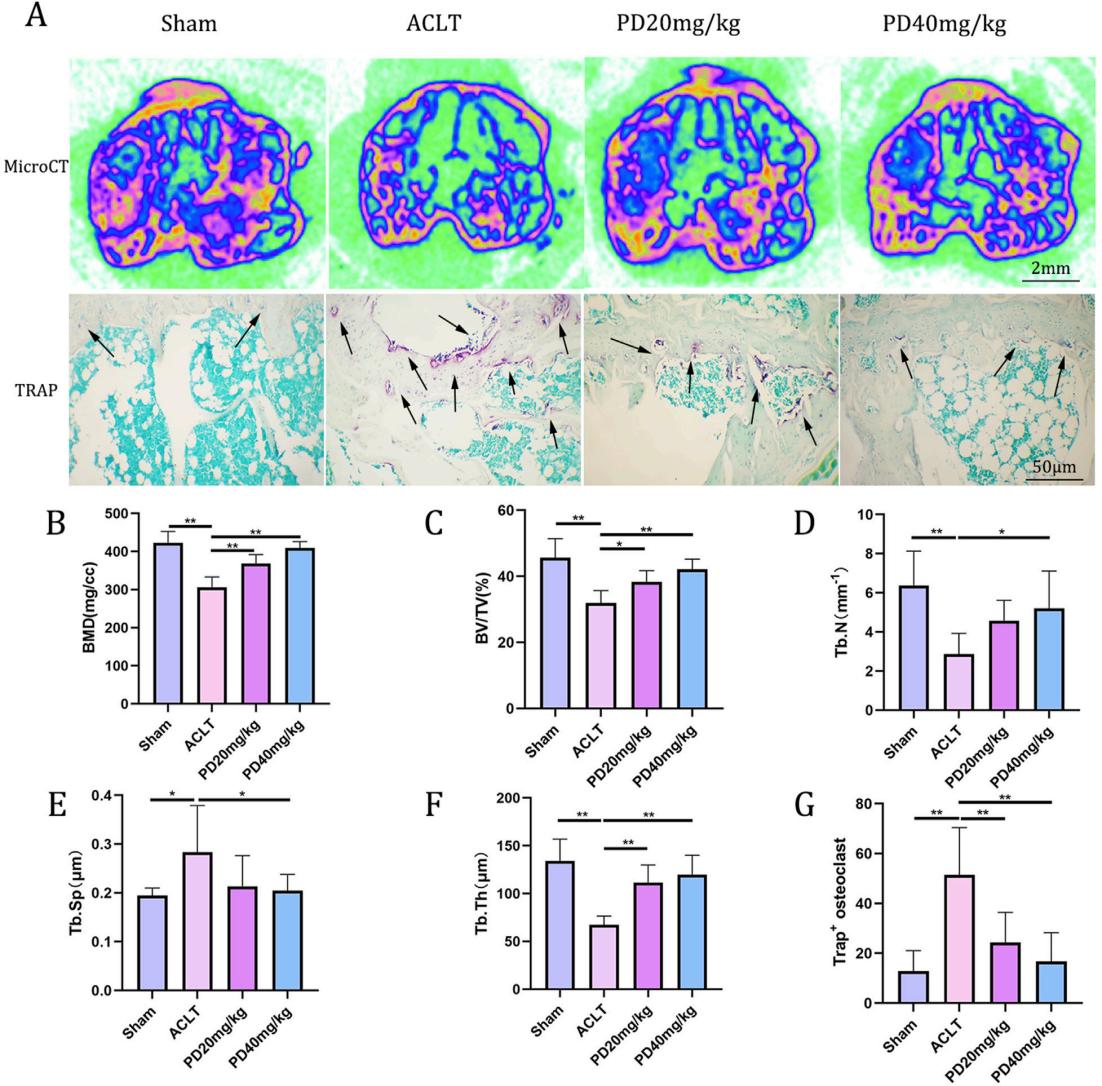
PD can regulate bone metabolism and improve the microstructure of knee subchondral bone trabeculae, thus delaying the progression of osteoarthritis. **(A)** Representative micro-CT image of tibial plateau subchondral bone among all groups. Tartrate acid phosphatase (TRAP) staining of tibial plateau subchondral bone. The osteoclasts were obtained by counting the number of TRAP-positive cells (N. Trap+). **(B–F)** The results of proximal tibia BMD, bone volume (BV)/total volume (TV), trabecular number (Tb.N; mm^−1^), trabecular thickness (Tb.Th; μm), and trabecular separation (Tb.Sp; μm) in different groups. **(G)** Quantitative analysis of TRAP-positive cells for tibial plateau subchondral bone. Note: *P < 0.05, **P < 0.01; scale bars = 2 mm as indicated.

**FIGURE 3:**
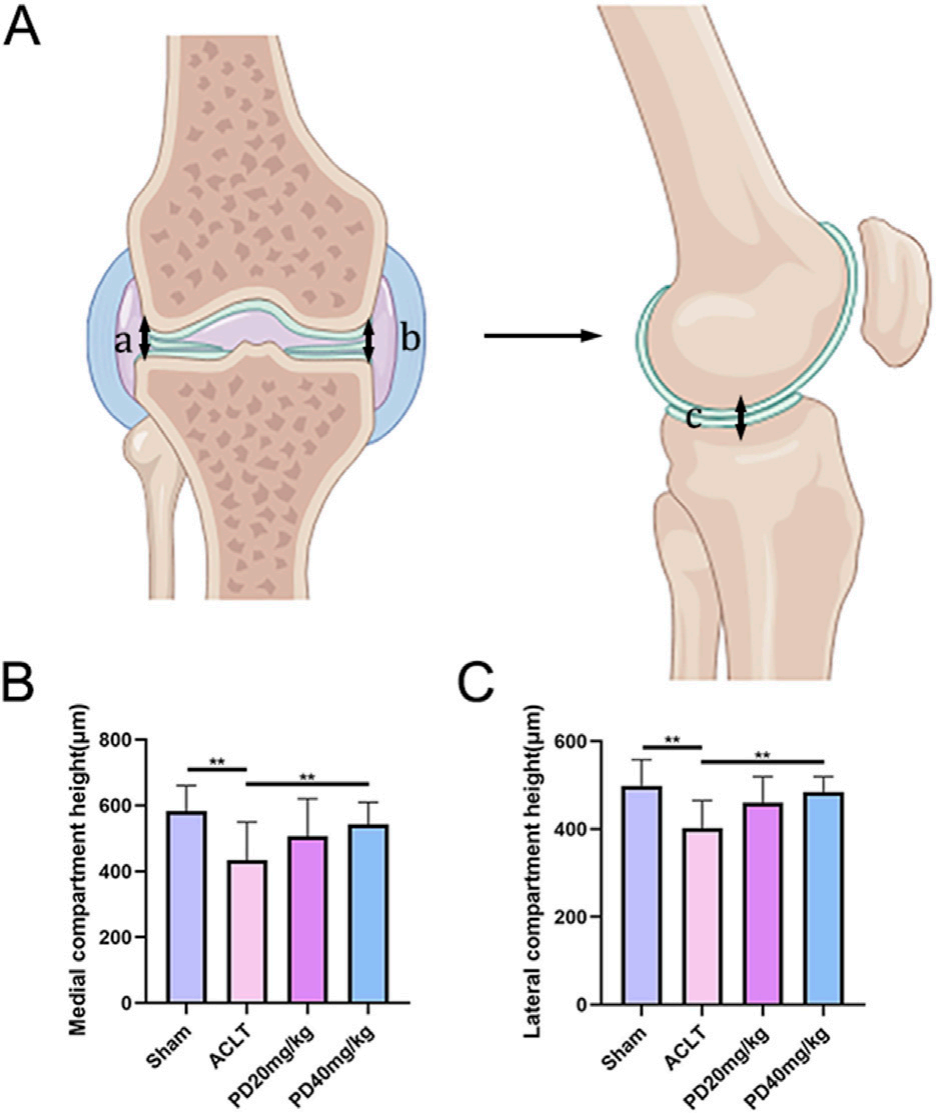
PD can maintain the knee space and delay the progression of osteoarthritis. **(A)** Schematic diagram of lateral and medial compartment height measurement of the knee joint. **(B, C)** The results of lateral and medial compartment height measurement in all groups. Note: *P < 0.05, **P < 0.01.

To delve deeper into the protective effects of PD on the metabolic and remodeling processes of subchondral bone in OA mice, MicroCT analysis was performed on samples from each group. The Sham group displayed well-organized trabecular bones with elevated bone density. In contrast, the ACLT group showed disorganized subchondral bone trabeculae, coupled with osteoporosis of varying severity. Following PD intervention, there was a notable enhancement in bone density, with significant improvements observed in bone trabecular assessment parameters, including BMD, BV/TV, Tb.Th, and Tb.N, while Tb.Sp was reduced. Remarkably, TRAP staining indicated a pronounced increase in osteoclast formation within the subchondral bone and at the cartilage-subchondral bone junction in the ACLT group relative to the Sham group; however, PD treatment led to a substantial reduction in the number of positive osteoclasts (Figure 2). Furthermore, the reduction or disappearance of the knee joint space is a critical pathological feature of osteoarthritis. We assessed the height of the lateral, medial, and central joint spaces in the sagittal plane for each group, which corresponds to the distance between the femur and tibia, using CT scans. The findings revealed that joint height was restored in a dose-dependent fashion following PD intervention when compared to the ACLT group (Figure 3). Collectively, these results imply that PD could potentially mitigate the progression of ACLT-induced osteoarthritis in mice by curbing the deterioration of subchondral bone trabecular microarchitecture, attenuating the reduction in joint height, and regulating bone metabolic activities.

### 3.3 Polydatin inhibits IL-1β-induced chondrocyte necroptosis and matrix degradation

**FIGURE 4:**
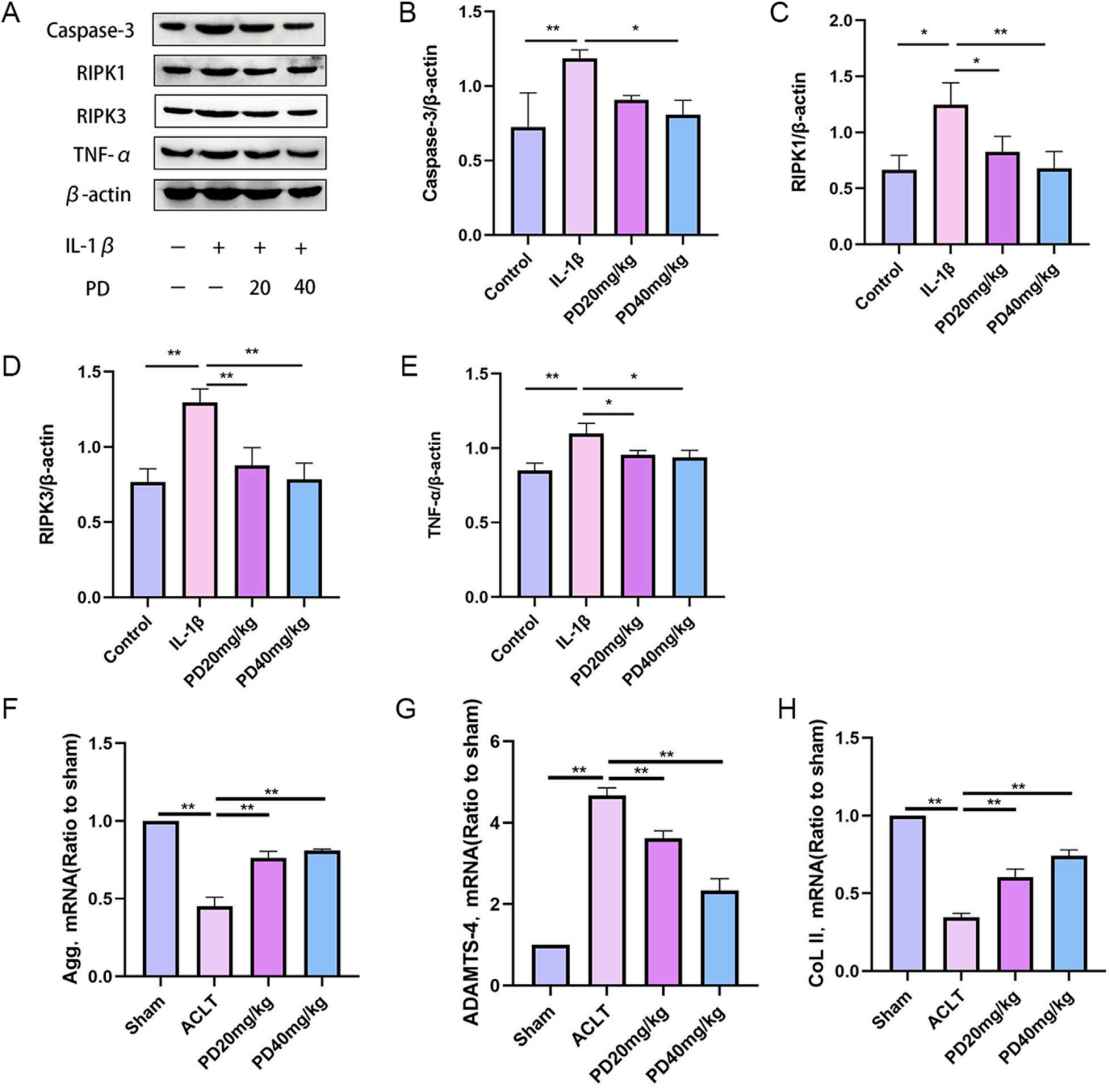
Polydatin inhibits IL-1β-induced chondrocyte necroptosis and matrix degradation *in vitro*. **(A–E)** Western blot and quantitative analysis of necroptosis-related markers (Cleaved caspase-3, PIPK1, PIPK3) and inflammatory factors (TNF-α) in chondrocytes cells treated with different doses of PD. **(F–H)** RT-qPCR analysis of Aggrecan, ADAMTS-4 and ColII in chondrocytes treated with different doses of PD. Note: *P < 0.05, **P < 0.01.

It is widely recognized that chondrocyte necrosis and apoptosis, along with dysregulation of the extracellular matrix metabolism, are pivotal pathological factors that expedite the progression of OA. In our study, we observed that IL-1β markedly upregulated the expression of proteins associated with necrosis and apoptosis (caspase3, PIPK1, PIPK3) and inflammatory cytokines (TNF-α). Notably, PD was capable of reversing these expression patterns in a dose-dependent manner, as illustrated in Figure 4. In terms of evaluating the metabolism of the extracellular matrix in articular chondrocytes, our findings confirmed that IL-1β significantly suppressed the expression of matrix-related proteins, such as collagen type II (col2) and Aggrecan, while concurrently increasing the expression of ADAMT4. PD intervention effectively mitigated the IL-1β-induced degradation of the extracellular matrix by enhancing the expression of Col2 and Aggrecan, and concurrently reducing the expression of ADAMT4, in a dose-dependent fashion, as depicted in Figure 4. These discoveries underscore the protective role of PD against IL-1β-induced cartilage damage associated with osteoarthritis *in vitro*, highlighting its potential therapeutic implications.

### 3.4 Polydatin effectively inhibits the activation of the NF-κB/ROS signaling pathway

**FIGURE 5:**
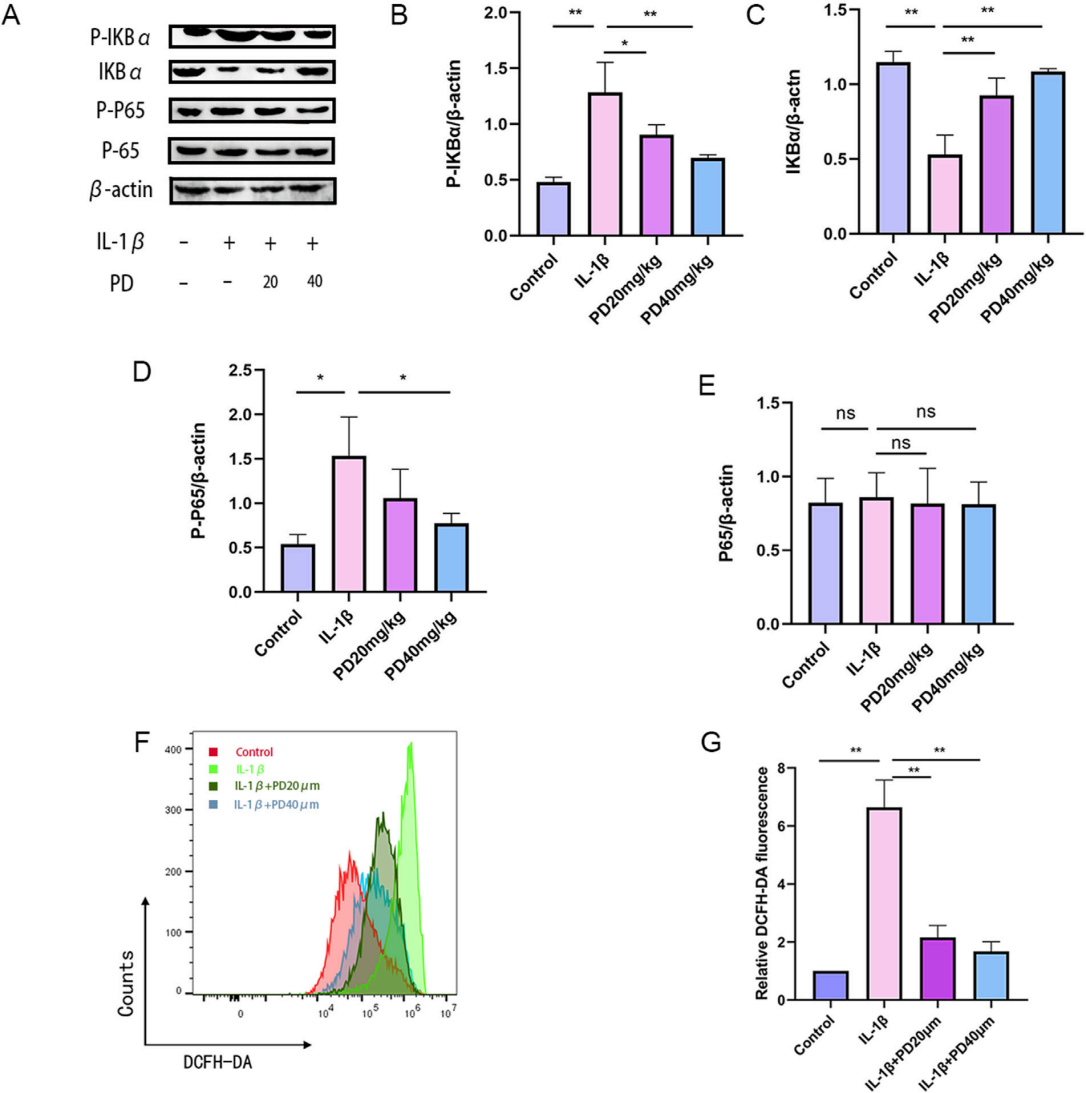
PD can significantly inhibit the accumulation of IL-1β-induced ROS by suppressing the NF-κB signaling pathway. **(A–E)** The expression levels of NF-κB signaling pathway-related proteins (p-IκBα, IκBα, p-p65, and p65) were detected by Western blot. **(F–G)** ROS levels were detected by flow cytometry in all groups. Note: *P < 0.05, **P < 0.01.

Elevated levels of reactive oxygen species (ROS) are known to markedly hasten the initiation and advancement of osteoarthritis (OA). In our investigation into the potential of PD to curb ROS generation in IL-1β-stimulated chondrocytes via modulation of the NF-κB signaling pathway, we noted a surge in ROS production following IL-1β stimulation. Encouragingly, PD was found to significantly ameliorate IL-1β-induced ROS accumulation in a dose-dependent fashion, as detailed in Figure 5.

Additionally, we assessed the phosphorylation status of key proteins within the NF-κB signaling axis, including p-IκBα, IκBα, p-p65, and p65. Our data revealed that IL-1β stimulation led to a pronounced upregulation of p-IκBα and p-p65, concurrent with a downregulation of IκBα. Importantly, PD effectively reversed these protein expression patterns in a dose-dependent manner, as depicted in Figure 5. Collectively, these findings suggest that PD can markedly suppress IL-1β-induced ROS accumulation by inhibiting the NF-κB signaling pathway.

**FIGURE 6:**
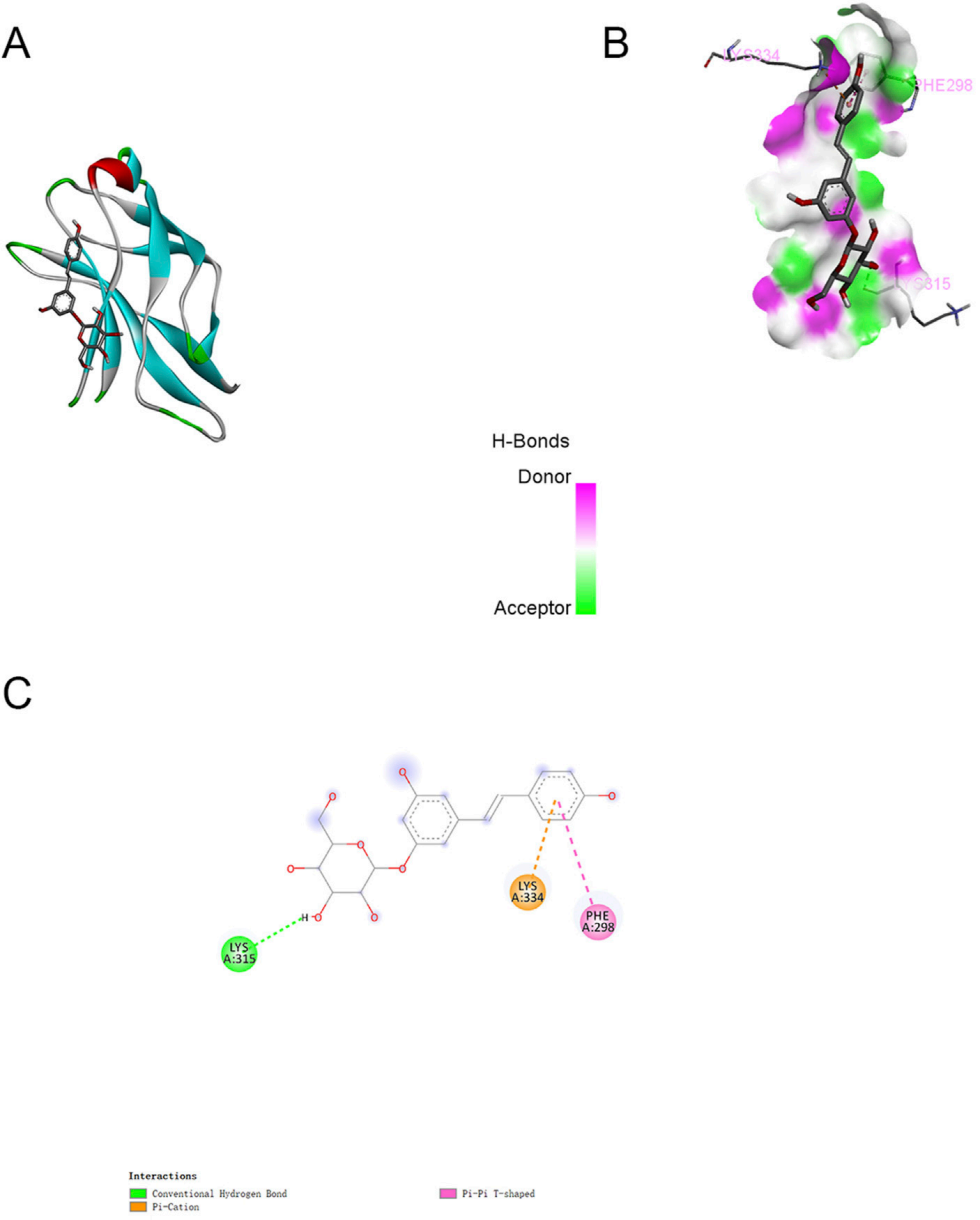
Molecular docking between PD and NF-κB (Typical docking mode and binding site images. **(A)**:The protein residues are represented by the ribbon model. **(B)**:The space filling model intuitively reflects the spatial position of PD in the inhibitory binding pocket of NF-κB. **(C)**:Docking modes and binding sites.

In parallel, we performed semi-flexible molecular docking analysis to elucidate the potential interactions between PD and NF-κB proteins, as illustrated in Figure 6. The docking studies indicated a binding energy of −6.7 kcal/mol, signifying a robust binding affinity of PD for the target protein, with lower energies correlating with stronger binding interactions. The complex formed by PD and NF-κB post-docking was visualized utilizing Pymol-2.1.0 software. The interaction between PD and NF-κB was primarily mediated through hydrogen bonds and hydrophobic interactions. Notably, PD was observed to form hydrogen bonds with VAL112, GLU17, and ILE69 of the NF-κB protein, with Pi-Anion engaging in a hydrogen bond with GLU17 and Pi-Alkyl with LEU140. Moreover, we delineated additional potential docking configurations and binding sites in Figure 6.

### 3.5 Polydatin inhibits IL-1β-induced macrophage M1 polarization

**FIGURE 7:**
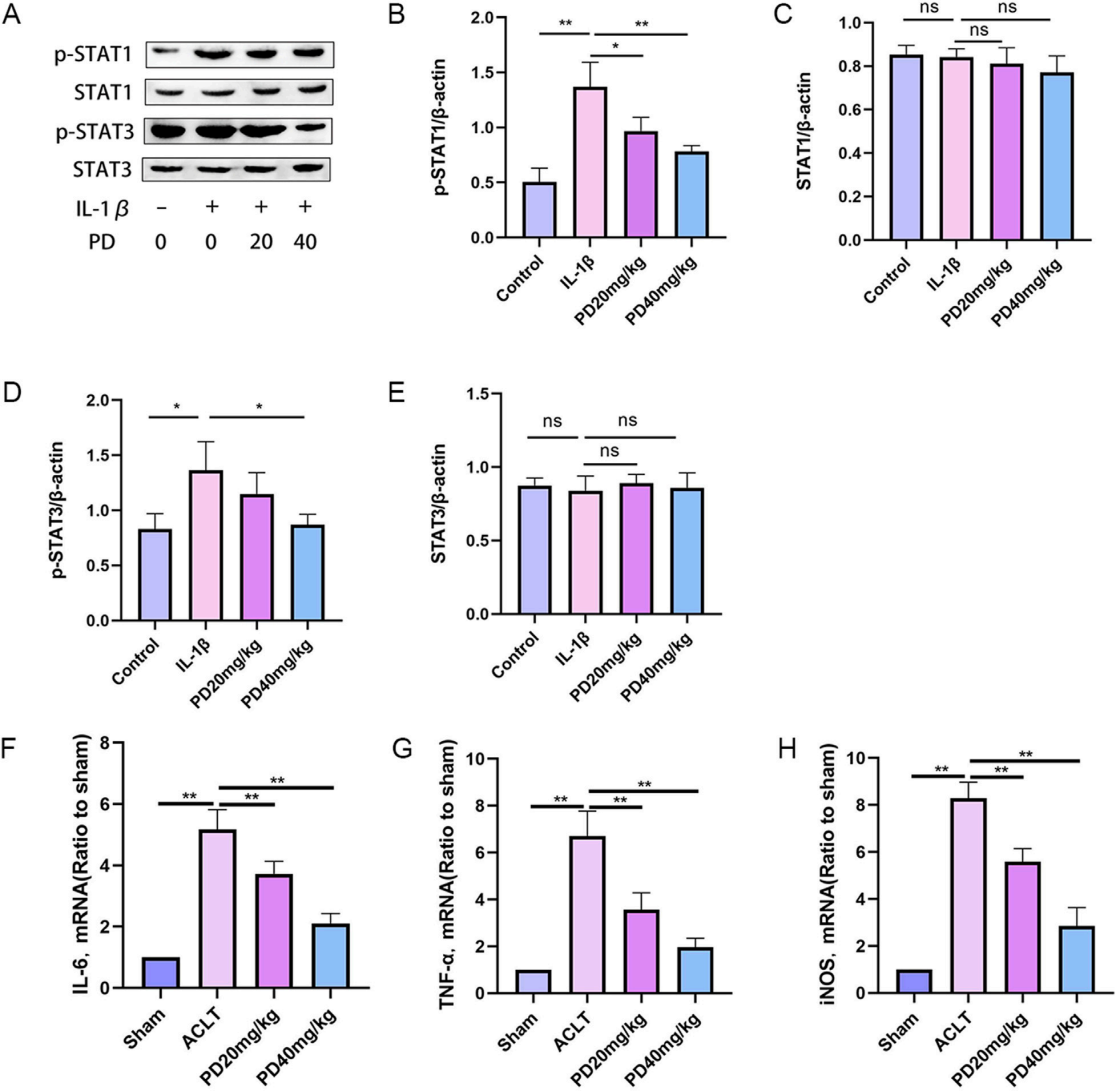
Polydatin inhibits IL-1β-induced macrophage M1 polarization. **(A–E)** The expression levels of repolarization-related markers (p-STAT1, STAT1, p-STAT3, and STAT3 proteins) were detected by Western blot. **(F–H)** M1 macrophage marker proteins (IL-6, TNF-α, and iNOS) were measured by RT-qPCR. Note: *P < 0.05, **P < 0.01.

The role of macrophage M1 polarization in the secretion of pro-inflammatory cytokines, which is accelerating the deterioration of articular cartilage, is gaining widespread interest among researchers. In our quest to elucidate the impact of PD on macrophage repolarization during the advancement of OA, we evaluated the expression levels of key markers associated with repolarization: p-STAT1, STAT1, p-STAT3, and STAT3. Our findings revealed that IL-1β substantially elevated the expression levels of p-STAT1 and p-STAT3 *in vitro*. However, PD intervention significantly diminished the IL-1β-induced phosphorylation of STAT1 and STAT3, as illustrated in Figure 7.

Furthermore, we quantified the expression of M1 macrophage signature proteins, including IL-6, TNF-α, and iNOS. The data demonstrated that PD effectively counteracted the upregulation of IL-6, TNF-α, and iNOS expression triggered by IL-1β in a dose-dependent manner, as depicted in Figure 7. Collectively, these findings suggest that PD has a significant inhibitory effect on macrophage M1 polarization induced in OA.

## 4 Discussion

OA is the most common chronic inflammatory joint disease. With the aging of the population, the incidence of osteoarthritis is gradually increasing, imposing a significant medical and economic burden on society. Osteoarthritis is characterized by cartilage degeneration, osteophyte formation, subchondral bone remodeling, and synovial hyperplasia (Kloppenburg, 2023). However, our understanding of the fundamental molecular mechanisms driving the pathogenesis of osteoarthritis is still limited, and we presently face a shortage of effective treatment strategies. Recently, the Bcr-Abl inhibitor DCC-2036 has been shown to significantly inhibit necrotic apoptosis and improve articular cartilage degeneration (Piao et al., 2023). Receptor-interacting protein 1 can regulate chondrocyte apoptosis under abnormal mechanical stress and suppress late-stage necrotic apoptosis to delay the development of temporomandibular joint osteoarthritis. Furthermore, injury-related molecular patterns promote the release of various inflammatory factors, recruiting macrophages, promoting local joint inflammation (Zhou et al., 2023). Therefore, a more comprehensive grasp of the regulation of necrotic apoptosis and inflammation in osteoarthritis will not only aid in shedding more light on the condition’s pathogenesis but also offer promising targets for its treatment.

PD have been demonstrated to have preventive and therapeutic effects for various diseases. For example, scientific research has revealed that PD have the capacity to ameliorate diabetic cardiomyopathy, harnessing their antioxidative, anti-inflammatory, and sustained-release attributes (Mostafa et al., 2021). PD act as mediators for the Xc-GSH-GPx4 axis and iron metabolism, effectively inhibiting ferroptosis and thereby mitigating cisplatin-induced acute kidney injury (Zhou et al., 2022). Furthermore, PD enhance insulin secretion, alleviate insulin resistance, regulate glucose and lipid metabolism, reduce hepatic lipid deposition, suppress inflammation and oxidative stress, lower uric acid accumulation, and modulate bone metabolism by influencing vital signaling pathways related to inflammation, oxidative stress, and cellular apoptosis (Tang et al., 2022; Luo et al., 2022; Yuan et al., 2022). Hecht et al. (2023) confirmed that early PD treatment mitigates joint degeneration and dampens pain in a mouse model of pseudoachondroplasia. In the context of our study, we delved into the potential mechanisms through which PD operate in osteoarthritis and affirmed that they proficiently mitigate damage to the articular cartilage in ACLT-induced osteoarthritic mice. Additionally, PD curtail necrotic apoptosis and inflammation by reducing the expression of caspase3, PIPK1, PIPK3, IL-1β, and TNF-α in chondrocytes. These findings underscore the protective role of PD in osteoarthritis.

Maladaptive apoptosis, a novel form of programmed cell death, represents a highly pro-inflammatory mode of cell demise. It is primarily initiated by TNF-α and transmits cell death signals through RIPK1 and RIPK3, ultimately acting on MLKL (Moriwaki et al., 2016). Following maladaptive apoptosis, cell membranes rupture, resulting in the release of a multitude of damage-associated molecules into the neighboring tissues. This, in turn, provokes inflammatory responses in the vicinity, consequently disturbing the equilibrium of the tissue microenvironment and further worsening the initiation and advancement of articular cartilage degeneration. Osteoarthritis is a complex, chronic metabolic disorder wherein inflammatory mediators are released by cartilage, bone, and synovium. These mediators are associated with the degradation of the extracellular matrix and the degeneration of cartilage and subchondral bone. Furthermore, cartilage cells undergo maturation and differentiation into hypertrophic chondrocytes, eventually leading to cartilage ossification, altering the structural components of joints and affecting biomechanical transmission—a critical factor in the development of osteoarthritis (Ying et al., 2023). In our study, we found that IL-1β significantly induces inflammation and maladaptive apoptosis in chondrocytes, consistent with previous research. Remarkably, the administration of PD significantly restrained IL-1β-triggered chondrocyte apoptosis. This was substantiated by a decrease in the expression of critical indicators, such as caspase3, PIPK1, and PIPK3, in addition to the suppression of inflammatory mediators (IL-6 and TNF-α) and components of the extracellular matrix (col2 and Agg). It has been reported that changes in the subchondral bone microenvironment, along with the interaction between osteoclasts and chondrocytes, are linked to osteoarthritis progression. Based on this, it is suggested that restoring abnormal subchondral bone remodeling and obstructing bridging H-type subchondral vessels can delay articular cartilage degeneration (Hu et al., 2021). Zhan et al. (Kloppenburg, 2023) confirmed that PD can not only promote osteogenic differentiation exhibit anti-osteoporotic activity, but also attenuates the severe sublesional bone loss in mice with chronic spinal cord injury. In our study, we also observed that the process of osteoarthritis is accompanied by abnormal subchondral bone metabolism. MicroCT scans confirmed that PD can effectively improve the microstructure of subchondral bone, including BMD, BV/TV, Tb.Th, Tb.N and Tb.Sp. Additionally, TRAP staining indicated that PD can regulate osteogenic differentiation in cartilage and subchondral bone, playing a vital role in maintaining joint bone metabolism balance.

Within chondrocytes isolated from osteoarthritis affected joints, the levels of inflammatory factors surge, concurrently activating the NF-κB signaling pathway. Upon the phosphorylation and degradation of IκBα, p65 undergoes activation through phosphorylation, translocating from the cytoplasm to the nucleus. This process initiates the transcription of downstream genes, effectively expediting the inflammatory response. Research has confirmed that activating the TLR2/NF-κB signaling pathway can increase the expression of inflammatory factors, while inhibiting this pathway’s activation effectively suppresses inflammation and improves OA (Yang et al., 2022). Wang et al. (2023) discovered that Eugenol can inhibit the phosphorylation of the NF-κB signaling pathway, subsequently modulating the secretion and expression levels of inflammation-related factors (IL-6, IL-8, and TNF-α) and extracellular matrix markers (aggrecan and collagen II). Ultimately, this plays a role in regulating the inflammatory response. To investigate the inhibitory effect of PD on the NF-κB signaling pathway and inflammation, we selected IL-1β as a positive control group and assessed the phosphorylation levels of NF-κB signaling pathway members and the expression of inflammation-related factors. The results indicated that PD can regulate the expression of inflammatory factors and ROS level by inhibiting the activation of the NF-κB signaling pathway.

The polarization of macrophages and their role in inflammation have garnered increasing attention. Macrophages display substantial diversity and adaptability, where M0 signifies unpolarized macrophages, M1 serves as pro-inflammatory mediators, and M2 fosters tissue healing and anti-inflammation, as elucidated in a pertinent study (Zhao et al., 2022). Macrophages polarize to the M1 state under inflammatory stimuli (including IFN-γ and TNF-α), exerting a potent pro-inflammatory effect (Martinez and Gordon, 2014). It has been reported that efficaciously ameliorating nonalcoholic fatty liver disease in mice entails diminishing the secretion of pro-inflammatory factors, reversing M1 polarization in macrophages, and augmenting M2 polarization, as detailed in a relevant study (Zhang et al., 2019). Dong et al. (2023) found that ASIC1a-CMPK2 can mediate M1 macrophage polarization and exacerbate chondrocyte senescence in osteoarthritis through IL-18. Furthermore, research suggests that NLRP3 (NACHT, LRR, and PYD domains-containing protein 3) as a downstream effector of ROS in M1-polarized macrophages is significantly inhibited after TRPV4 suppression, thereby delaying the progression of osteoarthritis (Sun et al., 2022). To investigate the role of PD in macrophage M1 polarization, we assessed the phosphorylation levels of the M1 polarization-associated markers STAT1 and STAT3. The results confirmed that PD significantly reduce the phosphorylation levels of p-STAT1 and p-STAT3 during IL-1β-induced M1 macrophage polarization, indicating that PD effectively inhibit IL-1β-induced M1 macrophage polarization. Simultaneously, PD also alleviate the elevation of IL-6, TNF-α, and iNOS induced by IL-1β.

**FIGURE 8:**
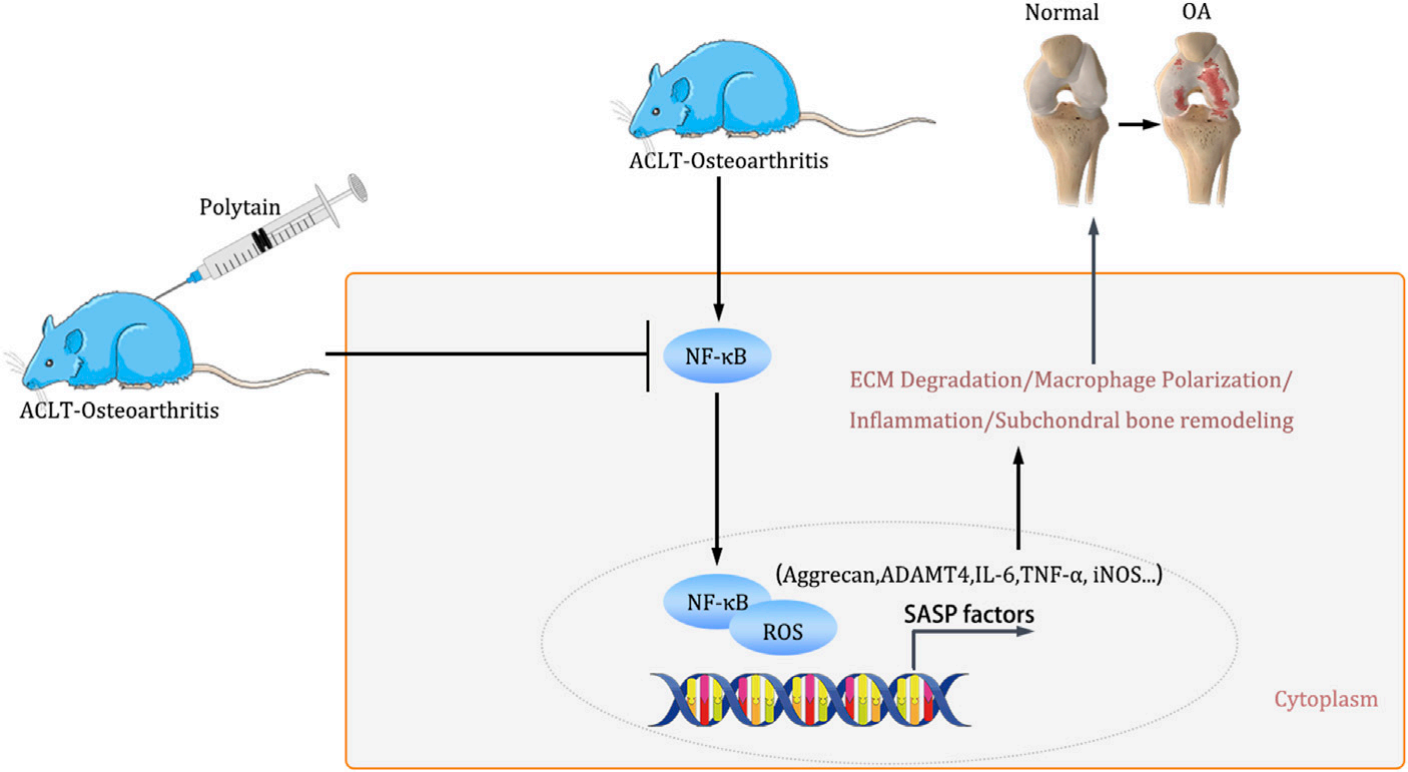
The schematic illustration for PD ameliorates inflammation response via NF-κB/ROS pathway in ACLT-induced mice osteoarthritis model.

In conclusion, our findings suggest that PD have the potential to mitigate the progression of ACLT-induced osteoarthritis by inhibiting extracellular matrix degradation and improving bone metabolism (Figure 8). Simultaneously, PD achieve the suppression of chondrocyte necrotic apoptosis and inflammation by inhibiting the activation of the NF-κB/ROS signaling pathway and macrophage M1 polarization. Our study indicates that PD may serve as a promising candidate for the treatment of osteoarthritis. However, our observations are still preliminary. Future studies should focus on the PD cycle, and controlled trials should be designed to evaluate the effects of PD in a time-dependent manner. We have included a discussion that acknowledges the limitations of our study, emphasizing that the PD dosages are tailored to animal experiments and do not have direct implications for clinical practice.

## Data Availability

The raw data supporting the conclusions of this article will be made available by the authors, without undue reservation.
