# Supplementary material for: Polydatin retards the progression of osteoarthritis by maintaining bone metabolicbalance and inhibiting macrophage polarization
Source: Front Bioeng Biotechnol. 2025 Jan 7;12:1514483. doi: 10.3389/fbioe.2024.1514483 (PMC11747576; doi:10.3389/fbioe.2024.1514483)
Supplement: Supplementary file 1 [file Presentation1.pdf]

frontiers

Author's proof

Before checking your proof, **please read the instructions below**

- Carefully read the entire proof and mark all corrections in the appropriate place, using the commenting tools (**Adobe Help**). Do not use the Edit tool, as direct edits could be missed (the PDF was blocked for editing to prevent this); annotate your corrections instead.
- Provide your corrections in a single PDF file or post your comments in the Production Forum, making sure to reference the relevant query/line number. Upload or post all your corrections directly in the Production Forum to avoid any comments being missed.
- We do not accept corrections via email or in the form of edited manuscripts.
- Do not provide scanned or handwritten corrections.
- Before you submit your corrections, make sure that you have checked your proof carefully, as once you approve it you won't be able to make any further corrections.
- To ensure timely publication of your article, please submit your corrections within 48 hours. We will inform you if we need anything else; do not contact us to confirm receipt.
- Note that the column alignment at the bottom of each page is not ensured during this Author's Proof stage. The columns will be correctly aligned in the final PDF publication. You may therefore notice small differences in the structure of the Author's Proof PDF versus the final publication.

Do you need help? Visit our **Production Help Center** for more information. If you can't find an answer to your question, contact your Production team directly by posting in the Production Forum.

NOTE FOR CHINESE-SPEAKING AUTHORS: If you'd like to see a Chinese translation, click on the symbol next to each query. **Only respond in English** as non-English responses will not be considered. Translated instructions for providing corrections can be found **here**.

Quick checklist

- ☐ **Author names** - Complete, accurate and consistent with your previous publications.
- ☐ **Affiliations** - Complete and accurate. Follow this style when applicable: Department, Institute, University, City, Country.
- ☐ **Tables** - Make sure the meaning/alignment of your Tables is correct with the applied formatting style.
- ☐ **Figures** - Make sure we are using the latest versions.
- ☐ **Funding and Acknowledgments** - List all relevant funders and acknowledgments.
- ☐ **Conflict of interest** - Ensure any relevant conflicts are declared.
- ☐ **Supplementary files** - Ensure the latest files are published and that no line numbers and tracked changes are visible. Also, the supplementary files should be cited in the article body text.
- ☐ **Queries** - You must reply to all **typesetter's queries below** in order for production to proceed.
- ☐ **Content** - Read all content carefully and ensure any necessary corrections are made, then **upload them** to the Production Forum.

Author queries form

Query no.	Details required	Author's response
Q1	Confirm that the article title is correct and check that it makes sense.	Yes

Query no.	Details required	Author's response
Q2	Provide a URL for the LOOP profile for the following authors if they wish this to be linked to the final published version. If they are not yet registered, ensure that they register with Frontiers at the provided link.<br>Xin-Yu Nan<br>Hui Wang<br>Shuo Pan<br>Gang Ji<br>Ya-Feng Guo<br>Ya-Heng Zhao<br>Gao-Cen Li<br>Shao-Shi Guo<br>Lu-Feng Lin<br>Yu-Jie Jin<br>Xue Li Zhang<br>Chang-Cheng Liu<br>Guo-bin Liu<br>If a URL is not provided, the profile link will not be added to the article. Non-registered authors and authors with profiles set to "Private" will have the default profile image displayed. Note that we will not be able to add profile links after publication.	Yes
Q3	The citation and surnames of all of the authors have been highlighted. Check that they are correct and consistent with your previous publications, and correct them if needed. Please note that this may affect the indexing of your article in repositories such as PubMed. If adding/removing authors, or changing the order of this list, please provide us with a signed Authorship Change form, which should be uploaded as a "Related Article" file type with your Author's Proof Corrections.	Yes
Q4	There is a discrepancy between the styling of the author names in the submission system and the manuscript. We have used [Xin-Yu Nan, Ya-Feng Guo, Ya-Heng Zhao, Gao-Cen Li, Shao-Shi Guo, Lu-Feng Lin, Yu-Jie Jin, Xue Li Zhang, Chang-Cheng Liu, Guo-Bin Liu] instead of [Yu Xin Nan, Feng Ya Guo, Heng Ya Zhao, Cen Gao Li, Shi Shao Guo, Feng Lu Lin, Jie Yu Jin, Li Xue Zhang, Cheng Chang Liu, Bin Guo Liu]. Please confirm that it is correct.	Yes
Q5	Provide the department details for Affiliation [1, 2].	1 The First Hospital of Hebei Medical University, No.89 Donggang Road, 050000, Shijiazhuang, Hebei, China.<br>2 Hebei Medical University, No. 361 Zhongshan East Road, 050000, Shijiazhuang, Hebei, China.
Q6	Confirm that all author affiliations are correctly listed. Per our style guidelines, affiliations are listed sequentially and follow author order. Requests for non-sequential affiliation listing will not be fulfilled. Note that affiliations should reflect those at the time during which the work was undertaken. If adding new affiliations, specify if these should be listed as a present address instead of a regular affiliation.	Yes
Q7	Confirm that the email address in your correspondence section is accurate. Any changes to corresponding authors requires individual confirmation from all original and added/removed corresponding authors.	Yes
Q8	Confirm that the keywords are correct, and keep them to a maximum of eight and a minimum of five. (Note: a keyword can be made up of one or more words.)	Yes
Q9	Check if the section headers (i.e., section leveling) have been correctly captured.	Yes
Q10	Confirm that the Data Availability statement is accurate. Note that this statement may have been amended to adhere to our Publication Ethics guidelines.	Yes
Q11	Confirm that the Ethics statement in this proof is correct. If this is not the latest version, please provide a revised Ethics statement. If your article contains identifiable human images, please check our Policies and Publication Ethics here.	Yes
Q12	Confirm that the details in the "Author Contributions" section are correct. If any contributions need to be added/edited, choose the appropriate CRedIT roles from the list available here and indicate which one(s) apply. Please be aware that writing roles ("Writing – original draft" and/or "Writing – review & editing") are a requirement for authorship.	In line 1368, change "ZX" to "X-LZ".

Query no.	Details required	Author's response
**Q13**	Ensure all grant numbers and funding information are included and accurate (after publication it is not possible to change this information). All funders should be credited, and all grant numbers should be correctly included in this section. If you provided a positive funding statement but don't provide funding details, then the statement will be updated to say no funding was received.	Yes
**Q14**	Confirm if the text included in the Conflict of Interest statement is correct. Please do not suggest edits to the wording of the final sentence, as this is standard for Frontiers' journal style, per our guidelines.	Yes
**Q15**	Ensure that any Supplementary Material is correctly published at this link: https://www.frontiersin.org/articles/10.3389/fbioe.2024.1514483/full#supplementary-material. Only published supplementary files will be available to download at the link. If the link does not work, you can check the file(s) directly in the Production Forum; the published supplementary files appear in green. We recommend citing all supplementary files. Please also provide captions for these files, if relevant. If you have any corrections, provide new files and we will publish them in the Forum. Frontiers will deposit ALL supplementary files to FigShare and they will receive a DOI. Notify us of any previously deposited material. If the Supplementary Material files contain identifiable or copyright images, please keep in mind that it is your responsibility, as the author, to ensure you have permission to use the images in the article. Please check this link for information on author responsibilities and the publication images.	Yes
**Q16**	Provide the DOI for "Hawker, 2019", if applicable.	10.4081/clr.2019.14780
**Q17**	Please verify the page range in "Zhou et al., 2022".	page：1-11
**Q18**	Ensure that all the figures, tables, and captions are correct, and that all figures are of the highest quality/resolution. You may upload improved figures to the Production Forum. If so, please describe in visual terms the exact changes(s) made to help us confirm that the updated version has been used in the finalized proof. Please note that figures and tables must be cited sequentially, per the author guidelines.	Yes
**Q19**	The image used in Figure 6 has part labels; however, the part labels are missing in the caption. Please either provide a revised figure or modify the caption, as necessary.	Molecular docking between PD and NF-κB (Typical docking mode and binding site images. A:The protein residues are represented by the ribbon model. B:The space filling model intuitively reflects the spatial position of PD in the inhibitory binding pocket of NF-κB Q19 .C: Docking modes and binding sites.
**Q20**	In given manuscript, there are 8 figure images, citations, and the captions was provided for figures 1 to 6 correctly. However, in the caption after figure 6 caption figure 8 and 9 present. So, we have checked the captions with their images and changed the figure 8 and 9 into figure 7 and 8. Please check and confirm.	Yes
**Q21**	If you decide to use previously published and/or copyrighted figures in your article, please keep in mind that it is your responsibility as the author to obtain the appropriate permissions and licenses to reproduce them, and to follow any citation instructions requested by third-party rights holders. If obtaining the reproduction rights involves the payment of a fee, these charges are to be paid by the authors.	Yes

TYPE Original Research
PUBLISHED XX XX 2024
DOI 10.3389/fbioe.2024.1514483

Check for updates

OPEN ACCESS

EDITED BY
Houyi Sun,
Shandong University, China

REVIEWED BY
Xianzuo Zhang,
Anhui Provincial Hospital, China
Xinyu Fang,
First Affiliated Hospital of Fujian Medical University, China

*CORRESPONDENCE
Chang-Cheng Liu,
✉ 57803022@hebmu.edu.cn
Guo-Bin Liu,
✉ liuguobin@hebmu.edu.cn

RECEIVED 21 October 2024
ACCEPTED 17 December 2024
PUBLISHED XX XX 2024

CITATION
Sun Q, Nan X-Y, Wang H, Pan S, Ji G, Guo Y-F, Zhao Y-H, Li G-C, Guo S-S, Lin L-F, Jin Y-J, Zhang XL, Liu C-C and Liu G-B (2024) Polydatin retards the progression of osteoarthritis by maintaining bone metabolicbalance and inhibiting macrophage polarization. *Front. Bioeng. Biotechnol.* 12:1514483. doi: 10.3389/fbioe.2024.1514483

COPYRIGHT
© 2024 Sun, Nan, Wang, Pan, Ji, Guo, Zhao, Li, Guo, Lin, Jin, Zhang, Liu and Liu. This is an open-access article distributed under the terms of the Creative Commons Attribution License (CC BY). The use, distribution or reproduction in other forums is permitted, provided the original author(s) and the copyright owner(s) are credited and that the original publication in this journal is cited, in accordance with accepted academic practice. No use, distribution or reproduction is permitted which does not comply with these terms.

Polydatin retards the progression of osteoarthritis by maintaining bone metabolicbalance and inhibiting macrophage polarization

Qi Sun[1], Xin-Yu Nan[2], Hui Wang[1], Shuo Pan[1], Gang Ji[1], Ya-Feng Guo[1], Ya-Heng Zhao[1], Gao-Cen Li[1], Shao-Shi Guo[1], Lu-Feng Lin[1], Yu-Jie Jin[1], Xue Li Zhang[1], Chang-Cheng Liu[1]* and Guo-Bin Liu[1]*

[1]The First Hospital of Hebei Medical University, Shijiazhuang, Hebei, China, [2]Hebei Medical University, Shijiazhuang, Hebei, China

Background: Polydatin (PD), also known as tiger cane glycoside, is a natural compound extracted from the Japanese knotweed plant, which is often referred to as white resveratrol. It exhibits anti-inflammatory, antioxidant, and anti-apoptotic effects in the treatment of various diseases. However, the potential molecular mechanisms of PD in osteoarthritis have not been clearly elucidated.

Methods: Anterior cruciate ligament transection (ACLT) surgery was performed to establish an osteoarthritis animal model. Female mice at the age of 12 weeks were intraperitoneally injected with different concentrations of PD (20 and 40 mg/kg). *In vitro* models were established by isolating mouse articular chondrocytes, which were subsequently treated with lipopolysaccharide or IL-1β for 24 h for subsequent experiments. In addition, different concentrations of PD were administered for 12 h. Morphological changes were observed by toluidine blue staining, joint bone metabolism changes were observed by tartrate-resistant acid phosphatase staining, immunohistochemistry was used to observe the expression levels of inflammatory factors and extracellular matrix. MicroCT analysis was conducted to assess changes in the microstructure of subchondral bone trabeculae, and Western blot was performed to measure the expression of nuclear factor kappa-light-chain-enhancer of activated B cells (NF-κB) signaling pathway and markers of M1 polarization in macrophages.

Results: PD significantly delays the progression of osteoarthritis induced by ACLT, effectively inhibits IL-1β-induced joint inflammation, bone metabolic remodeling and extracellular matrix degradation. In addition, paeoniflorin markedly suppresses the transmission of the NF-κB signaling pathway and reverses M1 polarization in macrophages induced by IL-1β.

Conclusion: Taken together, PD might be a potential therapeutic agent for the prevention and treatment of osteoarthritis.

KEYWORDS
Polydatin, cartilage, extracellular matrix, NF-κB, osteoarthritis

Q9

1 Introduction

Osteoarthritis (OA) is one of the most common chronic metabolic joint diseases worldwide, characterized by joint pain, morning stiffness, and loss of joint function. It affects more than 30% of individuals over the age of 65, with a higher prevalence in females than males (Hawker, 2019; Pan et al., 2022). Osteoarthritis often leads to disability, significantly diminishing patients' quality of life and increasing the economic and healthcare burden on their families and society. In general, the primary pathological features of Osteoarthritis include synovial inflammation, cartilage degeneration, and subchondral bone remodeling (Yue and Berman, 2022). Established risk factors for osteoarthritis encompass a wide range of factors, such as age, hormonal levels, gender, obesity, inflammation, genetics, oxidative stress, trauma, and various other elements (Wang et al., 2023; Driban et al., 2020). Aging and obesity have garnered increasing attention as contributing factors. With the global rise in obesity rates and the advent of an aging population, the number of osteoarthritis patients continues to grow (Yang et al., 2022; Hart et al., 2020). Unfortunately, the pathogenesis of osteoarthritis remains unclear, and effective therapeutic strategies to slow the progression of osteoarthritis are currently lacking. Therefore, gaining a better understanding of the potential molecular mechanisms of osteoarthritis is instrumental in identifying specific therapeutic targets for the treatment of osteoarthritis.

Unlike other tissues, chondrocytes are the sole cell type present in articular cartilage and serve as the primary regulatory units for cartilage metabolism (Piao et al., 2023). Under normal conditions, chondrocytes regulate both synthetic and catabolic processes in cartilage, concurrently secreting a significant amount of extracellular matrix to maintain joint homeostasis (Liu et al., 2022). Aberrations in chondrocyte metabolism are closely associated with phenotypes related to osteoarthritis, including inflammation, apoptosis, necrosis, and senescence, and may indeed be pivotal factors in the initiation and progression of the disease. Inflammation, as reported, plays a significant role in the pathogenesis of osteoarthritis, disrupting the homeostasis of the joint microenvironment, regulating bone metabolism through the NF-κB signaling pathway, and triggering downstream genes, including the conversion of pro-inflammatory cytokines such as IL-1β and IL-18 into mature pro-inflammatory factors, further exacerbating the progression of osteoarthritis (Shi et al., 2022; Sang et al., 2022; Wei et al., 2022). In the context of aerobic metabolism (oxidative phosphorylation) or the oxidation of nicotinamide adenine dinucleotide phosphate (NADPH), reactive oxygen species (ROS) are predominantly produced by the mitochondrial respiratory chain (Napolitano et al., 2021). It has been documented that there is a notable elevation of ROS levels within osteoarthritis tissues, which contributes to the progression of osteoarthritis by perturbing the cellular environment, including the upregulation of inflammatory factor expression (Collins et al., 2018). Moreover, the imbalance in extracellular matrix metabolism is another critical factor in the pathogenesis of osteoarthritis. High expression levels of MMP3, MMP13, and ADAMT4 in articular cartilage of osteoarthritis signify excessive extracellular matrix degradation, thereby accelerating the course of osteoarthritis (Lazarov et al., 2023).

Polydatin (PD), a natural and bioavailable derivative of the potent phytochemical quercetin, is known for its high bioavailability and its beneficial effects, including anti-inflammatory, antioxidant, and anti-apoptotic properties (Karami et al., 2022). Prior research has suggested that PD holds promise in the treatment of inflammation-related conditions such as diabetes, liver damage, cartilage degeneration, and dementia-related diseases (Yousef et al., 2021; Bao et al., 2022; Karami et al., 2022). Studies have demonstrated that PD can potentially address atherosclerosis through its anti-inflammatory, lipid metabolism-regulating, and antioxidative stress-alleviating mechanisms (Huang et al., 2021). Despite ongoing research exploring the potential protective role of resveratrol in osteoarthritis, the precise mechanisms underlying its protective effects in osteoarthritis remain incompletely understood.

In this study, we have demonstrated that PD primarily exerts its therapeutic effects by inhibiting inflammation, apoptosis, abnormal bone metabolism, and extracellular matrix degradation. Additionally, it achieves a significant reduction in Anterior cruciate ligament transection (ACLT) -induced OA by suppressing the activation of the NF-κB signaling pathway, which regulates macrophage polarization. Our findings highlight PD as an effective therapeutic modality for the treatment of osteoarthritis. To delve into the impact of PD on the advancement of OA, this research employs the ACLT method, which is widely accepted, to establish an OA model. Nevertheless, it is essential to recognize that this model has its constraints and may not comprehensively mirror the full range of pathological alterations characteristic of chronic degenerative knee osteoarthritis, particularly those related to hormonal fluctuations and the aging process.

2 Materials and methods

2.1 Animals

In this study, forty-eight 12-week-old healthy female C57BL/6J mice were purchased from Yi Wei Wo Technology Co., Ltd. (Shijiazhuang, China). The animals were group-housed four per cage under a 12-hour light-dark cycle with access to food and water at a constant temperature of 21°C. All experimental protocols were approved by the Institutional Animal Care and Use Committee. All procedures followed the guidelines of the Animal Care and Use Committee of the Hebei Medical University and admitted by the Animal Ethics Committee of the Hebei Medical University.

2.2 Experiment design and operation procedures

Adaptive feeding was conducted for one week, followed by a sham operation (A skin incision was made and then sutured) or anterior cruciate ligament transection (ACLT) on their right knees. All animals were given prophylactic antibiotics (penicillin-G; 40,000 U) for 3 days after the surgery. After ACLT surgery 3 days, the PD group was given intraperitoneal injection of PD 20 and 40 mg/kg/day for 12 weeks. The mice were weighted once a week, and the PD doses was adjusted accordingly. After the

treatment, the fresh serum was collected and the right knees were removed.

Half of the samples (n = 6, each group) were used for histological, immunohistochemical analysis. The other half underwent microcomputed tomography (μCT) scanning.

2.3 μCT testing

To investigate the alterations in the subchondral bone microarchitecture, fixed joint tissues were scanned with the SkyScan 1176 microcomputed tomography system. We define the trabecular area of subchondral bone under tibial plateau as regions of interest (ROI). We define the trabecular area of subchondral bone under tibial plateau as a ROI. NRecon v1.6 software was used to reconstruct images scanned at 50 kV, 450 μA, and 8 μm. Three-dimensional reconstructed images were obtained by with CTvox v3.0. The CTAn v1.14 and Data Viewer v.1.5 were used to select the ROI.

Transverse images of subchondral bone under tibial plateau were used to measure the microarchitecture after excluding the cortical bone. The joint height index (JHI) is calculated by measuring the height of both sides and the center of the joint space in the mid-sagittal plane for three times, which is a joint height measurement method proposed by our team. An overview of the subchondral bone structure under tibial plateau was obtained by measuring the bone mineral density (BMD), bone volume fraction (BV/TV), trabecular thickness (Tb.Th), trabecular number (Tb.N) and trabecular separation (Tb.Sp) parameters.

2.4 Histology and immunohistochemistry examinations

A two-month decalcification in 10% EDTA-2Na was performed after fixation in 10% neutral paraformaldehyde for 48 h. We dehydrated and embedded the paraffin-embedded decalcified samples. The sections were then cut into 6-μm slices for Toluidine Blue (TB) and immunohistochemistry staining. TB was performed using DB0057 per the manufacturers' directions to assess osteoarthritis-associated degeneration, such as structural disorganization, cartilage injury, and clefts. This analysis was performed by two independent researchers in a blinded manner.

2.5 Chondrocyte isolation and treatment

Articular cartilage was separated from the femoral heads of mice, and chondrocytes were separated as previously described. In short, the mice were killed and disinfected in 75% alcohol for 5 min. The entire articular cartilage was separated from the femoral heads and cut into pieces. 0.25% trypsin and 0.2% collagenase II were digested for 40 min and 4 h, respectively. Then, the cells were filtered through a 70 μm cell filter and washed three times with PBS. Subsequently, the collected chondrocytes were cultured in Dulbecco's Modified Eagle Medium (DMEM) at 37°C, 5% CO_2. The culture medium was changed every two to three days. The chondrocytes were used for subsequent experiments.

To induce inflammation and cell death by apoptosis, IL-1β (10 ng/mL; Sigma-Aldrich) was added to the culture medium and incubated for 24 h. Then, the cells were treated with different concentrations of PD (20, 40 μM) for 12 h, and the cells were collected for subsequent experiments.

2.6 Real-time polymerase chain reaction (RT-PCR) analysis

Samples for RT-PCR were obtained from the chondrocytes. A Gene Amp 7,700 Sequence Detection System (Applied Biosystems, Foster City, CA, United States) and SYBER® Premix Ex Taq™II kit (Takara, Kusatsu, Japan) were used for the RT-PCR. The primers for the selected genes are listed in Table 1. GAPDH was used as an endogenous control. The changes in relative mRNA transcript levels were calculated using the 2 (-Delta C(T)) method as previously described. The experiment was repeated at least three times to ensure accuracy.

2.7 Western blot analysis

The chondrocytes were collected and dissolved in a radioimmunoprecipitation analysis buffer using a cocktail of protease inhibitors. The protein lysate was electrophoresed with sodium dodecyl sulfate-polyacrylamide gel and transferred to nitrocellulose membranes. Next, 5% bovine serum albumin was used to block the membranes at 4°C for 2 h. The membranes were subsequently incubated with the following primary antibodies at 4°C overnight: Caspase3 (1:400, Abcam), RIPK1 (1:500, Abcam), RIPK3 (1:500, Abcam), TNF-α (1:200, Abcam) p-IκBα (1:400, GeneTex), IκBα(1:400, GeneTex), p-P65 (1:500, GeneTex), P65 (1:500, GeneTex), p-STAT1 (1:200, Abcam), STAT1 (1:200, Abcam), p-STAT3 (1:400, GeneTex), and STAT3 (1:400, GeneTex). After washing with TBST, the membranes were incubated with HRP-conjugated secondary antibodies at a dilution of 1:2000 at 4°C for 4 h. The proteins were visualized using an enhanced chemiluminescence kit (Merck Millipore, Billerica, MA, United States) and an imaging system (Bio Spectrum 600; UVP, Upland, CA, United States).

2.8 ROS detection

The intercellular ROS levels were detected by dichlorofluorescein fluorescence as previously described (Rastogi et al., 2010). Briefly, cells were treated by 5 ng/mL IL-1β for 24 h and then treated with different concentrations of PD (20 and 40 μM) for another 12 h. Subsequently, cells were incubated in 2.5 μM DCF in DMEM at 37°C in the dark for 30 min. The ROS level was detected by flow cytometry. The mean fluorescence intensities for DCF were analyzed by Flow-Jo (BD FACSCalibur; BD Biosciences).

TABLE 1 Sequences of primers used for RT-PCR.

Gene	Forward primer (5′-3′)	Reverse primers (5′-3′)
GAPDH	GGGGAGCCAAAAGGGTCATCATCT	GAGGGGCCATCCACAGTCTTCT
IL-6	GGCGGATCGGATGTTGTGAT	GGACCCCAGACAATCGGTTG
TNF-α	GGAACACGTCGTGGGATAATG	GGCAGACTTTGGATGCTTCTT
iNos	CTCTTCGACGACCCAGAAAAC	CAAGGCCATGAAGTGAGGCTT
Col II	TGTTTGCAGAGCACTACTTGAA	ACCAGGGGAACCACTCTCAC
Aggrecan	GAAGTGGCGTCCAAACCAAC	AGCTGGTAATTGCAGGGGAC
ADAMT4	CGTTCCGCTCCTGTAACACT	TTGAAGAGGTCGGTTCGGTG

2.9 Molecular modelling

The compound structure file (CID: 5281718) of the PD was downloaded from the Pubchem database (https://pubchem.ncbi.nlm.nih.gov/), and the PDB format structure file (PDB ID: 1u3j) of the protein IL37 was downloaded from the PDB database (https://www.rcsb.org/). Pymol-2.1.0 software was used to remove water molecules and small molecular ligands from the target, and AutoDock Tools-1.5.6 was used to hydrogenate and compute charge the protein and save it as pdbqt format. The energy of the compound is minimized using the PyRx built-in tool (OpenBabel). Proteins were used as receptors and compounds as ligands. vina 2.0 inside PyRx software was used to perform molecular docking, calculate binding energy and output result files. Finally, PyMol software was used to visualize the results. The Affinity (kcal/mol) value represents the binding capacity of the two, and the lower the binding capacity, the more stable the binding between the ligand and the receptor, adopt the Discovery Studio 2020 Client (https://discover.3ds.com/discovery-studio-visualizer-download) for visual analysis (The lower the binding energy, the better the binding).

2.10 Statistical analysis

The data in this study are presented as the mean ± standard deviation. SPSS 26.0 version was used for all statistical analyses. Normal distribution and homogeneity of variances were evaluated by Shapiro–Wilk and Bartlett's tests. One-way analysis of variance and Fisher's protected least significant difference test were adopted for analyzing significant differences. The Kruskal-Wallis test was used to analyze the histological scores.

3 Results

3.1 Polydatin alleviates damage to ACLT-induced osteoarthritic cartilage

In our study aimed at examining the protective effects of PD on OA, we developed an ACLT-induced OA model and treated OA mice with varying concentrations of PD (20 and 40 mg/kg/day over a 12-week period). Consistent with our expectations, Toluidine Blue staining demonstrated that the ACLT group showed pronounced cartilage degeneration compared to the Sham group, characterized by a marked increase in glycosaminoglycan depletion within the extracellular matrix and more advanced cartilage erosion. Post-PD intervention, however, we observed an enhancement in the smoothness of the cartilage surface, with chondrocytes appearing more numerous, well-filled and orderly arranged. Furthermore, PD was found to notably slow the progression of ACLT-induced OA in a dose-dependent fashion, a finding corroborated by our scoring data presented in Figure 1. In addition, we evaluated the articular cartilage expression levels of inflammatory cytokines IL-6 and TNF-α across all groups. Our results indicated a significant decrease in IL-6 and TNF-α expression in the articular cartilage of PD-treated mice when compared to the ACLT group, as depicted in Figure 1. Collectively, these observations suggest that PD has the potential to suppress the advancement of ACLT-induced OA by modulating the inflammatory response.

3.2 Polydatin improves the stability of subchondral bone trabecular microstructure in ACLT-induced osteoarthritis

To delve deeper into the protective effects of PD on the metabolic and remodeling processes of subchondral bone in OA mice, MicroCT analysis was performed on samples from each group. The Sham group displayed well-organized trabecular bones with elevated bone density. In contrast, the ACLT group showed disorganized subchondral bone trabeculae, coupled with osteoporosis of varying severity. Following PD intervention, there was a notable enhancement in bone density, with significant improvements observed in bone trabecular assessment parameters, including BMD, BV/TV, Tb.Th, and Tb.N, while Tb.Sp was reduced. Remarkably, TRAP staining indicated a pronounced increase in osteoclast formation within the subchondral bone and at the cartilage-subchondral bone junction in the ACLT group relative to the Sham group; however, PD treatment led to a substantial reduction in the number of positive osteoclasts (Figure 2). Furthermore, the reduction or disappearance of the knee joint space is a critical pathological feature of osteoarthritis. We assessed the height of the lateral, medial, and central joint spaces in the sagittal plane for each group, which corresponds to the distance between the femur and tibia, using CT scans. The findings revealed that joint height was restored in a dose-dependent fashion following PD intervention when compared to the

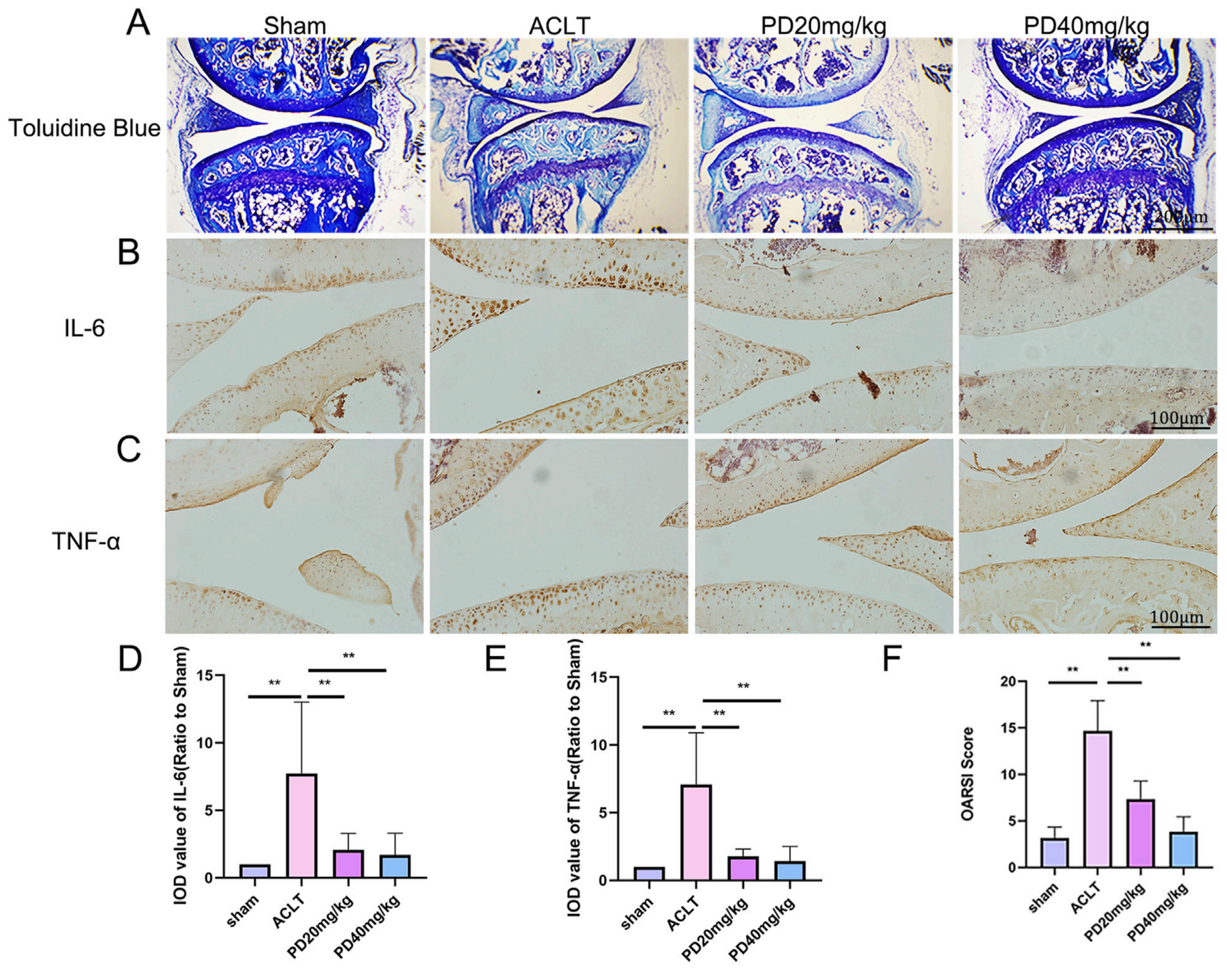

FIGURE 1
PD attenuated cartilage destruction and inflammatory development in ACLT mice. After ACLT surgery for 3 days, mice were intraperitoneally injected with different concentrations of PD (20 and 40 mg/kg) and maintained for twelve weeks. **(A)** Representative images of Toluidine Blue in knee joints. Immunohistochemistry assay for **(B)** IL-6 and **(C)** TNF-α in the knee joints among all groups. **(D, E)** Immunohistological analysis showed that the expression of IL-6 and TNF-α in the ACLT group was greater than that in the PD groups. **(F)** The quantification of Osteoarthritis Research Society International (OARSI) score in different groups. Note: $^{*}P < 0.05$, $^{**}P < 0.01$.

ACLT group (Figure 3). Collectively, these results imply that PD could potentially mitigate the progression of ACLT-induced osteoarthritis in mice by curbing the deterioration of subchondral bone trabecular microarchitecture, attenuating the reduction in joint height, and regulating bone metabolic activities.

3.3 Polydatin inhibits IL-1β-induced chondrocyte necroptosis and matrix degradation

It is widely recognized that chondrocyte necrosis and apoptosis, along with dysregulation of the extracellular matrix metabolism, are pivotal pathological factors that expedite the progression of OA. In our study, we observed that IL-1β markedly upregulated the expression of proteins associated with necrosis and apoptosis (caspase3, PIPK1, PIPK3) and inflammatory cytokines (TNF-α). Notably, PD was capable of reversing these expression patterns in a dose-dependent manner, as illustrated in Figure 4. In terms of evaluating the metabolism of the extracellular matrix in articular chondrocytes, our findings confirmed that IL-1β significantly suppressed the expression of matrix-related proteins, such as collagen type II (col2) and Aggrecan, while concurrently increasing the expression of ADAMT4. PD intervention effectively mitigated the IL-1β-induced degradation of the extracellular matrix by enhancing the expression of Col2 and Aggrecan, and concurrently reducing the expression of ADAMT4, in a dose-dependent fashion, as depicted in Figure 4. These discoveries underscore the protective role of PD against IL-1β-induced cartilage damage associated with osteoarthritis *in vitro*, highlighting its potential therapeutic implications.

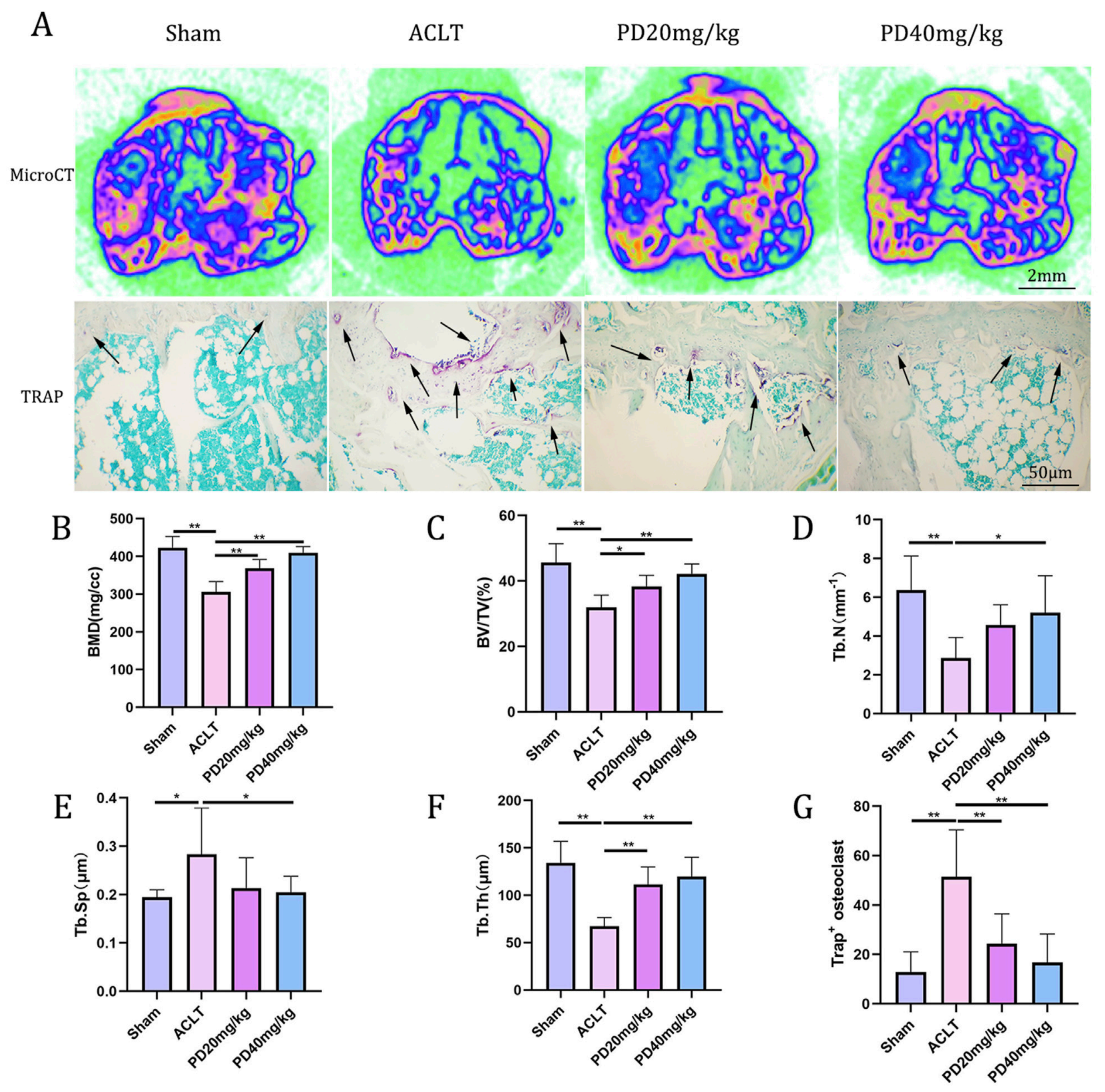

FIGURE 2
PD can regulate bone metabolism and improve the microstructure of knee subchondral bone trabeculae, thus delaying the progression of osteoarthritis. **(A)** Representative micro-CT image of tibial plateau subchondral bone among all groups. Tartrate acid phosphatase (TRAP) staining of tibial plateau subchondral bone. The osteoclasts were obtained by counting the number of TRAP-positive cells (N. Trap+). **(B–F)** The results of proximal tibia BMD, bone volume (BV)/total volume (TV), trabecular number (Tb.N; mm^{-1}), trabecular thickness (Tb.Th; μm), and trabecular separation (Tb.Sp; μm) in different groups. **(G)** Quantitative analysis of TRAP-positive cells for tibial plateau subchondral bone. Note: $*P < 0.05$, $**P < 0.01$; scale bars = 2 mm as indicated.

3.4 Polydatin effectively inhibits the activation of the NF-κB/ROS signaling pathway

Elevated levels of reactive oxygen species (ROS) are known to markedly hasten the initiation and advancement of osteoarthritis (OA). In our investigation into the potential of PD to curb ROS generation in IL-1β-stimulated chondrocytes via modulation of the NF-κB signaling pathway, we noted a surge in ROS production following IL-1β stimulation. Encouragingly, PD was found to significantly ameliorate IL-1β-induced ROS accumulation in a dose-dependent fashion, as detailed in Figure 5.

Additionally, we assessed the phosphorylation status of key proteins within the NF-κB signaling axis, including p-IκBα, IκBα, p-p65, and p65. Our data revealed that IL-1β stimulation led to a pronounced upregulation of p-IκBα and p-p65, concurrent with a downregulation of IκBα. Importantly, PD effectively reversed these protein expression patterns in a dose-dependent manner, as depicted in Figure 5. Collectively, these findings suggest that PD

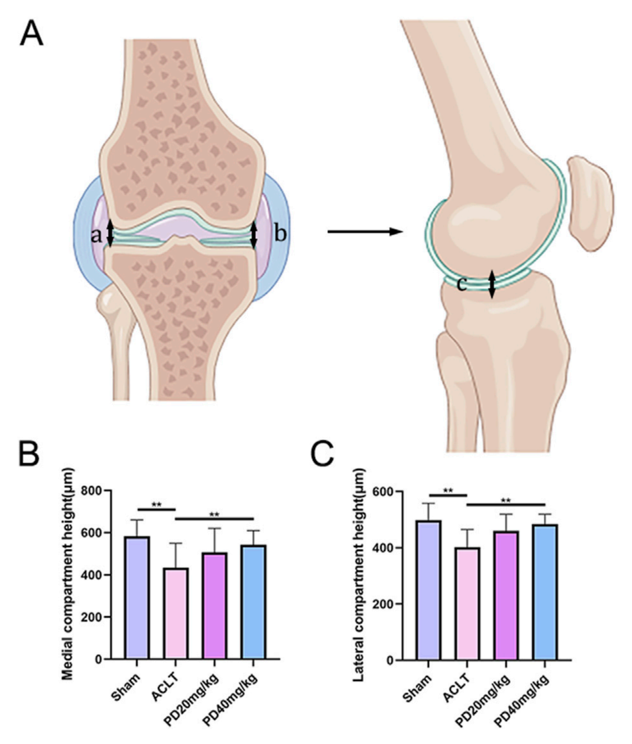

FIGURE 3
PD can maintain the knee space and delay the progression of osteoarthritis. **(A)** Schematic diagram of lateral and medial compartment height measurement of the knee joint. **(B, C)** The results of lateral and medial compartment height measurement in all groups. Note: $*P < 0.05$, $**P < 0.01$.

can markedly suppress IL-1β-induced ROS accumulation by inhibiting the NF-κB signaling pathway.

In parallel, we performed semi-flexible molecular docking analysis to elucidate the potential interactions between PD and NF-κB proteins, as illustrated in Figure 6. The docking studies indicated a binding energy of −6.7 kcal/mol, signifying a robust binding affinity of PD for the target protein, with lower energies correlating with stronger binding interactions. The complex formed by PD and NF-κB post-docking was visualized utilizing Pymol-2.1.0 software. The interaction between PD and NF-κB was primarily mediated through hydrogen bonds and hydrophobic interactions. Notably, PD was observed to form hydrogen bonds with VAL112, GLU17, and ILE69 of the NF-κB protein, with Pi-Anion engaging in a hydrogen bond with GLU17 and Pi-Alkyl with LEU140. Moreover, we delineated additional potential docking configurations and binding sites in Figure 6.

3.5 Polydatin inhibits IL-1β-induced macrophage M1 polarization

The role of macrophage M1 polarization in the secretion of pro-inflammatory cytokines, which is accelerating the deterioration of articular cartilage, is gaining widespread interest among researchers. In our quest to elucidate the impact of PD on macrophage repolarization during the advancement of OA, we evaluated the expression levels of key markers associated with repolarization: p-STAT1, STAT1, p-STAT3, and STAT3. Our findings revealed that IL-1β substantially elevated the expression levels of p-STAT1 and p-STAT3 *in vitro*. However, PD intervention significantly diminished the IL-1β-induced phosphorylation of STAT1 and STAT3, as illustrated in Figure 7.

Furthermore, we quantified the expression of M1 macrophage signature proteins, including IL-6, TNF-α, and iNOS. The data demonstrated that PD effectively counteracted the upregulation of IL-6, TNF-α, and iNOS expression triggered by IL-1β in a dose-dependent manner, as depicted in Figure 7. Collectively, these findings suggest that PD has a significant inhibitory effect on macrophage M1 polarization induced in OA.

4 Discussion

OA is the most common chronic inflammatory joint disease. With the aging of the population, the incidence of osteoarthritis is gradually increasing, imposing a significant medical and economic burden on society. Osteoarthritis is characterized by cartilage degeneration, osteophyte formation, subchondral bone remodeling, and synovial hyperplasia (Kloppenburg, 2023). However, our understanding of the fundamental molecular mechanisms driving the pathogenesis of osteoarthritis is still limited, and we presently face a shortage of effective treatment strategies. Recently, the Bcr-Abl inhibitor DCC-2036 has been shown to significantly inhibit necrotic apoptosis and improve articular cartilage degeneration (Piao et al., 2023). Receptor-interacting protein 1 can regulate chondrocyte apoptosis under abnormal mechanical stress and suppress late-stage necrotic apoptosis to delay the development of temporomandibular joint osteoarthritis. Furthermore, injury-related molecular patterns promote the release of various inflammatory factors, recruiting macrophages, promoting local joint inflammation (Zhou et al., 2023). Therefore, a more comprehensive grasp of the regulation of necrotic apoptosis and inflammation in osteoarthritis will not only aid in shedding more light on the condition's pathogenesis but also offer promising targets for its treatment.

PD have been demonstrated to have preventive and therapeutic effects for various diseases. For example, scientific research has revealed that PD have the capacity to ameliorate diabetic cardiomyopathy, harnessing their antioxidative, anti-inflammatory, and sustained-release attributes (Mostafa et al., 2021). PD act as mediators for the Xc-GSH-GPx4 axis and iron metabolism, effectively inhibiting ferroptosis and thereby mitigating cisplatin-induced acute kidney injury (Zhou et al., 2022). Furthermore, PD enhance insulin secretion, alleviate insulin resistance, regulate glucose and lipid metabolism, reduce hepatic lipid deposition, suppress inflammation and oxidative stress, lower uric acid accumulation, and modulate bone metabolism by influencing vital signaling pathways related to inflammation, oxidative stress, and cellular apoptosis (Tang et al., 2022; Luo et al., 2022; Yuan et al., 2022). Hecht et al. (2023) confirmed that early PD treatment mitigates joint degeneration and dampens pain in a mouse model of pseudoachondroplasia. In the context of our study, we delved into the potential mechanisms through which PD operate in osteoarthritis and affirmed that they proficiently mitigate damage to the articular cartilage in ACLT-induced osteoarthritic

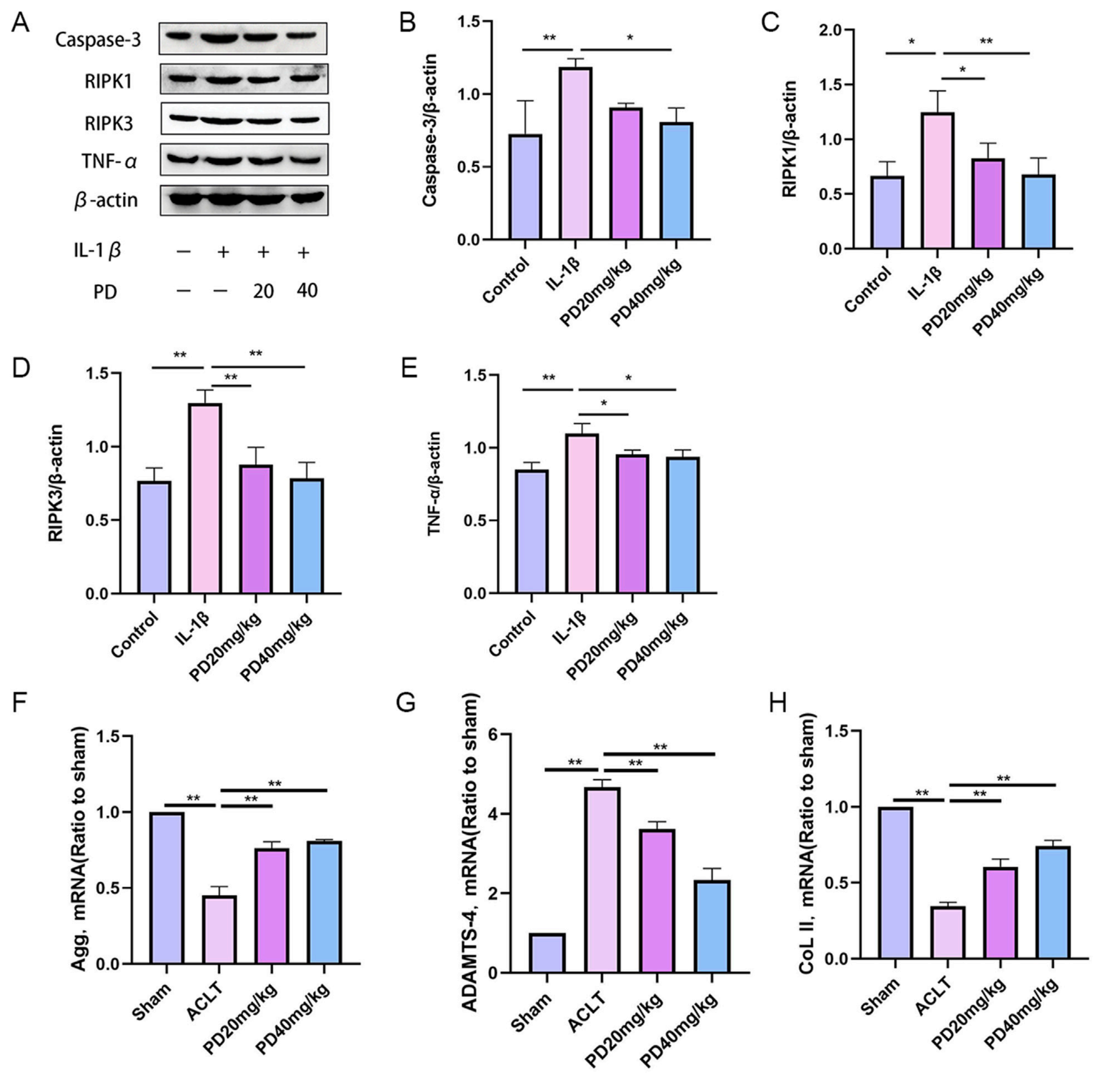

FIGURE 4
Polydatin inhibits IL-1β-induced chondrocyte necroptosis and matrix degradation *in vitro*. **(A–E)** Western blot and quantitative analysis of necroptosis-related markers (Cleaved caspase-3, PIPK1, PIPK3) and inflammatory factors (TNF-α) in chondrocytes cells treated with different doses of PD. **(F–H)** RT-qPCR analysis of Aggrecan, ADAMTS-4 and ColII in chondrocytes treated with different doses of PD. Note: $*P < 0.05$, $**P < 0.01$.

mice. Additionally, PD curtail necrotic apoptosis and inflammation by reducing the expression of caspase3, PIPK1, PIPK3, IL-1β, and TNF-α in chondrocytes. These findings underscore the protective role of PD in osteoarthritis.

Maladaptive apoptosis, a novel form of programmed cell death, represents a highly pro-inflammatory mode of cell demise. It is primarily initiated by TNF-α and transmits cell death signals through RIPK1 and RIPK3, ultimately acting on MLKL (Moriwaki et al., 2016). Following maladaptive apoptosis, cell membranes rupture, resulting in the release of a multitude of damage-associated molecules into the neighboring tissues. This, in turn, provokes inflammatory responses in the vicinity, consequently disturbing the equilibrium of the tissue microenvironment and further worsening the initiation and advancement of articular cartilage degeneration. Osteoarthritis is a complex, chronic metabolic disorder wherein inflammatory mediators are released by cartilage, bone, and synovium. These mediators are associated with the degradation of the extracellular matrix and the degeneration of cartilage and subchondral bone. Furthermore, cartilage cells undergo maturation and differentiation into hypertrophic chondrocytes, eventually leading to cartilage ossification, altering the structural components of joints and affecting biomechanical transmission—a critical factor in the development of osteoarthritis (Ying et al., 2023). In our study, we

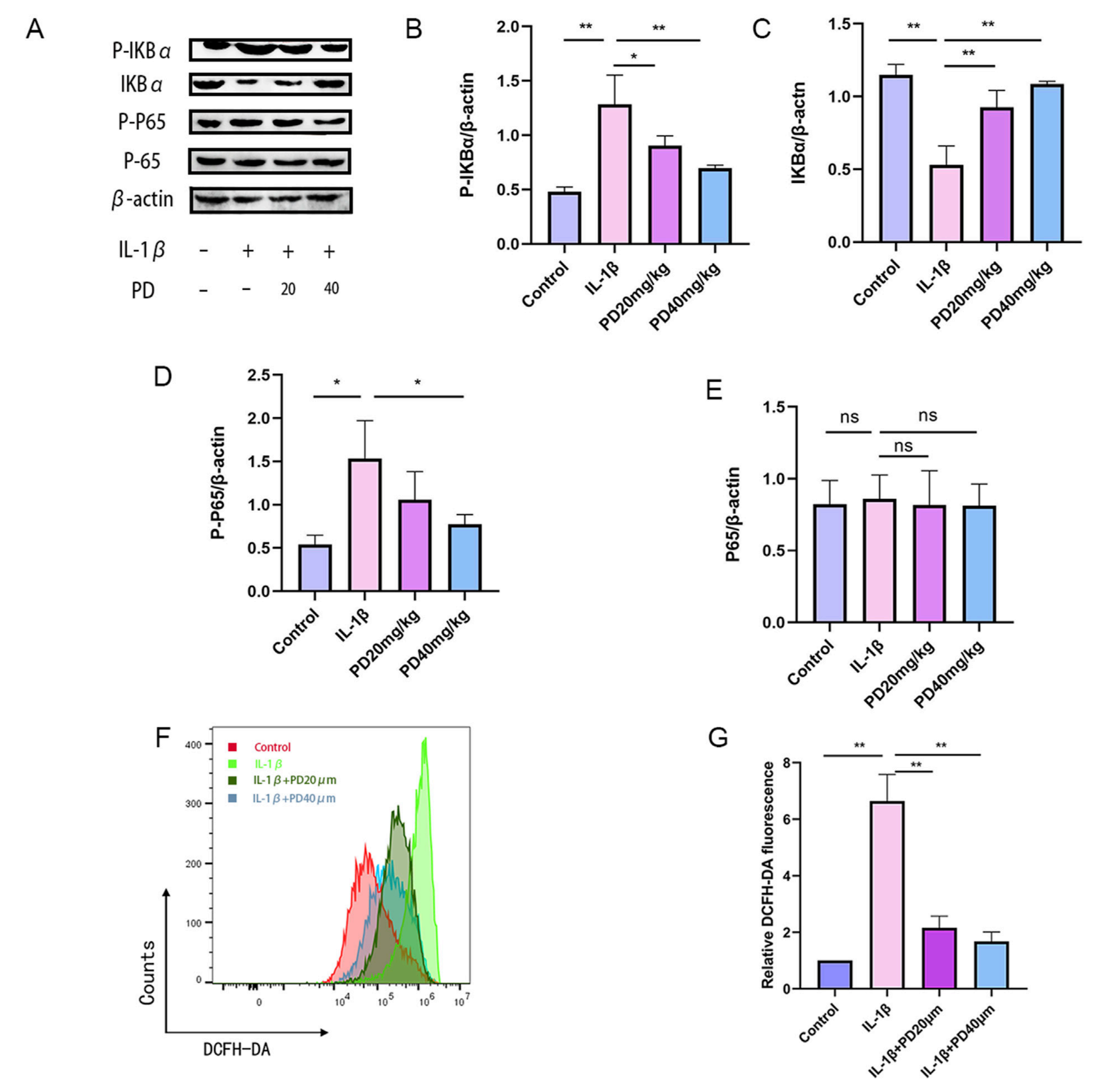

FIGURE 5
PD can significantly inhibit the accumulation of IL-1β-induced ROS by suppressing the NF-κB signaling pathway. **(A–E)** The expression levels of NF-κB signaling pathway-related proteins (p-IκBα, IκBα, p-p65, and p65) were detected by Western blot. **(F–G)** ROS levels were detected by flow cytometry in all groups. Note: $*P < 0.05$, $**P < 0.01$.

found that IL-1β significantly induces inflammation and maladaptive apoptosis in chondrocytes, consistent with previous research. Remarkably, the administration of PD significantly restrained IL-1β-triggered chondrocyte apoptosis. This was substantiated by a decrease in the expression of critical indicators, such as caspase3, PIPK1, and PIPK3, in addition to the suppression of inflammatory mediators (IL-6 and TNF-α) and components of the extracellular matrix (col2 and Agg). It has been reported that changes in the subchondral bone microenvironment, along with the interaction between osteoclasts and chondrocytes, are linked to osteoarthritis progression. Based on this, it is suggested that restoring abnormal subchondral bone remodeling and obstructing bridging H-type subchondral vessels can delay articular cartilage degeneration (Hu et al., 2021). Zhan et al. (Kloppenburg, 2023) confirmed that PD can not only promote osteogenic differentiation exhibit anti-osteoporotic activity, but also attenuates the severe sublesional bone loss in mice with chronic spinal cord injury. In our study, we also observed that the process of osteoarthritis is accompanied by abnormal subchondral bone metabolism. MicroCT scans confirmed that PD can effectively improve the microstructure of subchondral bone, including BMD, BV/TV, Tb.Th, Tb.N and Tb.Sp. Additionally, TRAP staining indicated that PD can regulate osteogenic differentiation in cartilage and subchondral

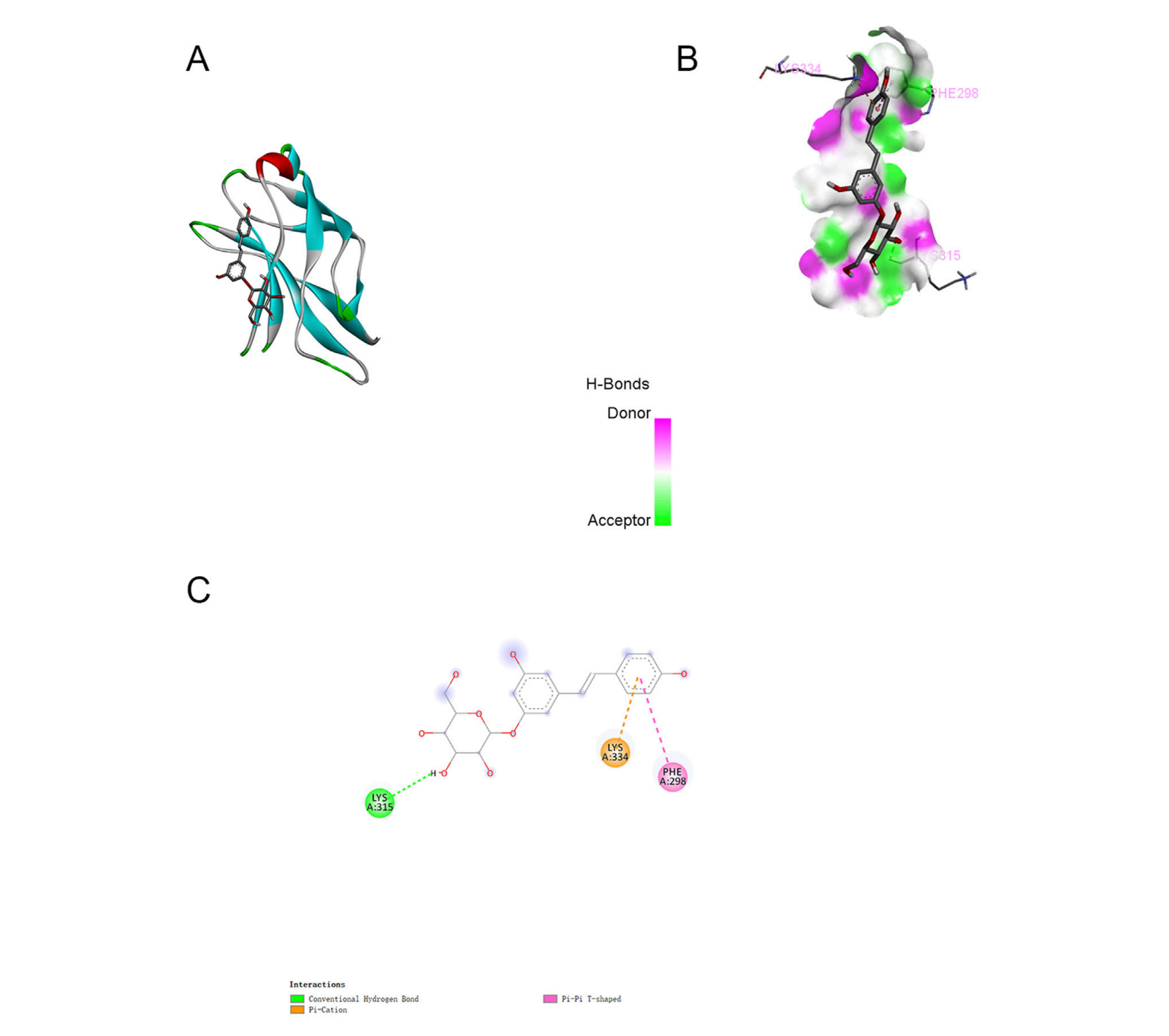

FIGURE 6
Q19 Molecular docking between PD and NF-κB (Typical docking mode and binding site images). The protein residues are represented by the ribbon model. The space filling model intuitively reflects the spatial position of PD in the inhibitory binding pocket of NF-κB.

bone, playing a vital role in maintaining joint bone metabolism balance.

Within chondrocytes isolated from osteoarthritis affected joints, the levels of inflammatory factors surge, concurrently activating the NF-κB signaling pathway. Upon the phosphorylation and degradation of IκBα, p65 undergoes activation through phosphorylation, translocating from the cytoplasm to the nucleus. This process initiates the transcription of downstream genes, effectively expediting the inflammatory response. Research has confirmed that activating the TLR2/NF-κB signaling pathway can increase the expression of inflammatory factors, while inhibiting this pathway's activation effectively suppresses inflammation and improves OA (Yang et al., 2022). Wang et al. (2023) discovered that Eugenol can inhibit the phosphorylation of the NF-κB signaling pathway, subsequently modulating the secretion and expression levels of inflammation-related factors (IL-6, IL-8, and TNF-α) and extracellular matrix markers (aggrecan and collagen II). Ultimately, this plays a role in regulating the inflammatory response. To investigate the inhibitory effect of PD on the NF-κB signaling pathway and inflammation, we selected IL-1β as a positive control group and assessed the phosphorylation levels of NF-κB signaling pathway members and the expression of inflammation-related factors. The results indicated that PD can regulate the expression of inflammatory factors and ROS level by inhibiting the activation of the NF-κB signaling pathway.

The polarization of macrophages and their role in inflammation have garnered increasing attention. Macrophages display substantial diversity and adaptability, where M0 signifies unpolarized macrophages, M1 serves as pro-inflammatory mediators, and M2 fosters tissue healing and anti-inflammation, as elucidated in a pertinent study (Zhao et al., 2022). Macrophages polarize to the M1 state under inflammatory stimuli (including IFN-γ and TNF-α),

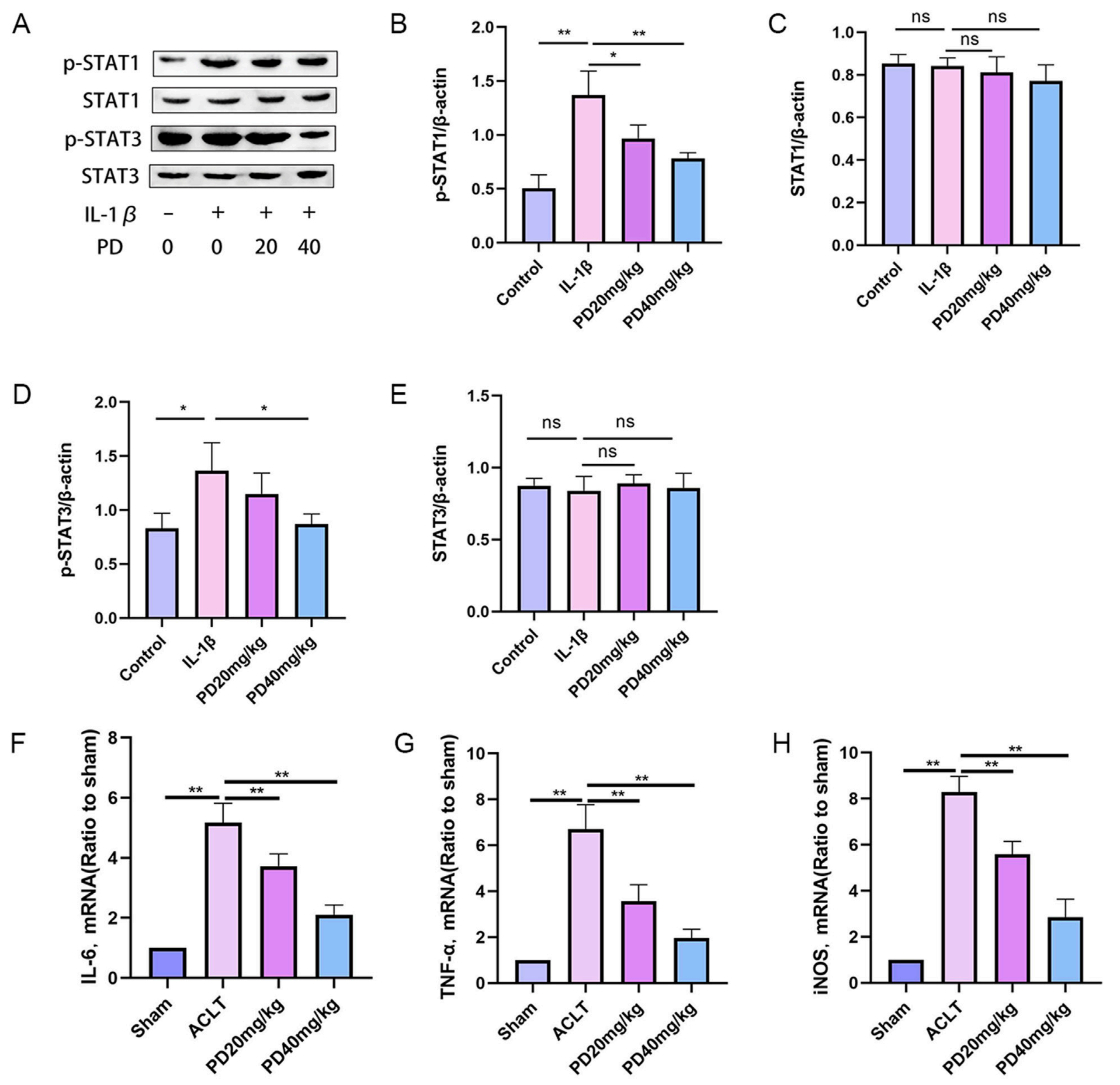

FIGURE 7
Polydatin inhibits IL-1β-induced macrophage M1 polarization. **(A–E)** The expression levels of repolarization-related markers (p-STAT1, STAT1, p-STAT3, and STAT3 proteins) were detected by Western blot. **(F–H)** M1 macrophage marker proteins (IL-6, TNF-α, and iNOS) were measured by RT-qPCR. Note: $*P < 0.05$, $**P < 0.01$.

Q20

exerting a potent pro-inflammatory effect (Martinez and Gordon, 2014). It has been reported that efficaciously ameliorating nonalcoholic fatty liver disease in mice entails diminishing the secretion of pro-inflammatory factors, reversing M1 polarization in macrophages, and augmenting M2 polarization, as detailed in a relevant study (Zhang et al., 2019). Dong et al. (2023) found that ASIC1a-CMPK2 can mediate M1 macrophage polarization and exacerbate chondrocyte senescence in osteoarthritis through IL-18. Furthermore, research suggests that NLRP3 (NACHT, LRR, and PYD domains-containing protein 3) as a downstream effector of ROS in M1-polarized macrophages is significantly inhibited after TRPV4 suppression, thereby delaying the progression of osteoarthritis (Sun et al., 2022). To investigate the role of PD in macrophage M1 polarization, we assessed the phosphorylation levels of the M1 polarization-associated markers STAT1 and STAT3. The results confirmed that PD significantly reduce the phosphorylation levels of p-STAT1 and p-STAT3 during IL-1β-induced M1 macrophage polarization, indicating that PD effectively inhibit IL-1β-induced M1 macrophage polarization. Simultaneously, PD also alleviate the elevation of IL-6, TNF-α, and iNOS induced by IL-1β.

In conclusion, our findings suggest that PD have the potential to mitigate the progression of ACLT-induced osteoarthritis by inhibiting extracellular matrix degradation and improving bone

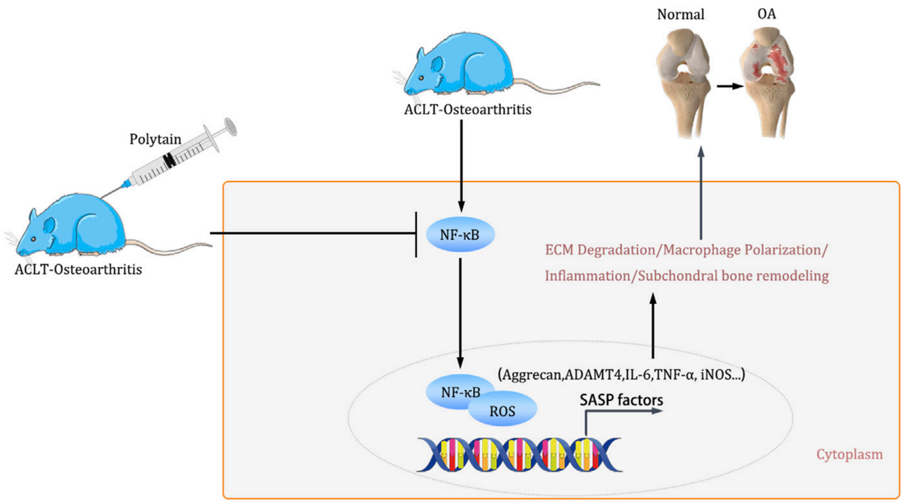

FIGURE 8
The schematic illustration for PD ameliorates inflammation response via NF-κB/ROS pathway in ACLT-induced mice osteoarthritis model.

metabolism (Figure 8). Simultaneously, PD achieve the suppression of chondrocyte necrotic apoptosis and inflammation by inhibiting the activation of the NF-κB/ROS signaling pathway and macrophage M1 polarization. Our study indicates that PD may serve as a promising candidate for the treatment of osteoarthritis. However, our observations are still preliminary. Future studies should focus on the PD cycle, and controlled trials should be designed to evaluate the effects of PD in a time-dependent manner. We have included a discussion that acknowledges the limitations of our study, emphasizing that the PD dosages are tailored to animal experiments and do not have direct implications for clinical practice.

Data availability statement

Q10

The raw data supporting the conclusions of this article will be made available by the authors, without undue reservation.

Ethics statement

Q11

The animal studies were approved by Laboratory Animal Ethical and Welfare Committee of Hebei Medical University. The studies were conducted in accordance with the local legislation and institutional requirements. Written informed consent was obtained from the owners for the participation of their animals in this study.

Author contributions

Q12

QS: Data curation, Funding acquisition, Investigation, Project administration, Writing–original draft, Formal Analysis, Methodology, Resources, Software, Validation, Visualization. X-YN: Conceptualization, Data curation, Investigation, Methodology, Project administration, Writing–original draft, Software, Supervision, Validation. HW: Conceptualization, Project administration, Supervision, Writing–original draft. SP: Data curation, Formal Analysis, Investigation, Writing–original draft. GJ: Investigation, Methodology, Writing–original draft, Conceptualization, Data curation, Supervision. Y-FG: Methodology, Writing–original draft, Investigation, Validation. Y-HZ: Formal Analysis, Methodology, Writing–original draft, Conceptualization. G-CL: Formal Analysis, Methodology, Writing–original draft, Project administration, Software. S-SG: Data curation, Methodology, Writing–original draft, Formal Analysis, Investigation. L-FL: Conceptualization, Data curation, Writing–original draft, Methodology, Validation. Y-JJ: Conceptualization, Data curation, Investigation, Supervision, Writing–original draft, Formal Analysis, Software. XZ: Conceptualization, Data curation, Investigation, Supervision, Writing–original draft. C-CL: Conceptualization, Data curation, Funding acquisition, Investigation, Methodology, Project administration, Resources, Writing–review and editing. G-BL: Conceptualization, Funding acquisition, Investigation, Methodology, Project administration, Resources, Supervision, Writing–review and editing.

Funding

The author(s) declare that financial support was received for the research, authorship, and/or publication of this article. This work was supported by the Hebei Provincial Health Commission Youth Science and Technology Project, Other topics of Hebei Provincial Health Commission (No: 20240936 and 20241239). Q13

Conflict of interest

The authors declare that the research was conducted in the absence of any commercial or financial relationships that could be construed as a potential conflict of interest. Q14

Generative AI statement

The author(s) declare that no Generative AI was used in the creation of this manuscript.

Publisher's note

All claims expressed in this article are solely those of the authors and do not necessarily represent those of their affiliated organizations, or those of the publisher, the editors and the reviewers. Any product that may be evaluated in this article, or claim that may be made by its manufacturer, is not guaranteed or endorsed by the publisher.

Supplementary material

The Supplementary Material for this article can be found online at: https://www.frontiersin.org/articles/10.3389/fbioe.2024.1514483/full#supplementary-material

References

Bao, H. L., Chen, C. Z., Ren, C. Z., Sun, K. Y., Liu, H., Song, S. H., et al. (2022). Fu ZR: polydatin ameliorates hepatic ischemia-reperfusion injury by modulating macrophage polarization. *Hepatobiliary Pancreat. Dis. Int.* 23, 25–34. doi:10.1016/j.hbpd.2022.08.009

Collins, J. A., Diekman, B. O., and Loeser, R. F. (2018). Targeting aging for disease modification in osteoarthritis. *Curr. Opin. Rheumatol.* 30 (1), 101–107. doi:10.1097/BOR.0000000000000456

Dong, L., Zhao, Y., Sun, C., Ou Yang, Z., Chen, F., Hu, W., et al. (2023). ASIC1a-CMPK2-mediated M1 macrophage polarization exacerbates chondrocyte senescence in osteoarthritis through IL-18. *Int. Immunopharmacol.* 124 (Pt A), 110878. doi:10.1016/j.intimp.2023.110878

Driban, J. B., Bannuru, R. R., Eaton, C. B., Spector, T. D., Hart, D. J., McAlindon, T. E., et al. (2020). The incidence and characteristics of accelerated knee osteoarthritis among women: the Chingford cohort. *BMC Musculoskelet. Disord.* 21 (1), 60. doi:10.1186/s12891-020-3073-3

Hart, H. F., van Middelkoop, M., Stefanik, J. J., Crossley, K. M., and Bierma-Zeinstra, S. (2020). Obesity is related to incidence of patellofemoral osteoarthritis: the Cohort Hip and Cohort Knee (CHECK) study. *Rheumatol. Int.* 40 (2), 227–232. doi:10.1007/s00296-019-04472-9

Hawker, G. A. (2019). Osteoarthritis is a serious disease. *Clin. Exp. Rheumatol.* 37 (Suppl. 120), 3–6.

Hecht, J. T., Veerisetty, A. C., Patra, D., Hossain, M. G., Chiu, F., Mobed, C., et al. (2023). Early resveratrol treatment mitigates joint degeneration and dampens pain in a mouse model of pseudoachondroplasia (PSACH). *Biomolecules* 13 (10), 1553. doi:10.3390/biom13101553

Hu, W., Chen, Y., Dou, C., and Dong, S. (2021). Microenvironment in subchondral bone: predominant regulator for the treatment of osteoarthritis. *Ann. Rheum. Dis.* 80 (4), 413–422. doi:10.1136/annrheumdis-2020-218089

Huang, L., He, S., Cai, Q., Li, F., Wang, S., Tao, K., et al. (2021). Polydatin alleviates traumatic brain injury: role of inhibiting ferroptosis. *Biochem. Biophys. Res. Commun.* 556, 149–155. doi:10.1016/j.bbrc.2021.03.108

Karami, A., Fakhri, S., Kooshki, L., and Khan, H. (2022). Polydatin: pharmacological mechanisms, therapeutic targets, biological activities, and Health benefits. *Molecules* 27 (19), 6474. doi:10.3390/molecules27196474

Kloppenburg, M. (2023). Inflammation is a relevant treatment target in osteoarthritis. *Lancet* 402, 1725–1726. doi:10.1016/S0140-6736(23)01726-9

Lazarov, T., Juarez-Carreno, S., Cox, N., and Geissmann, F. (2023). Physiology and diseases of tissue-resident macrophages. *Nature* 618 (7966), 698–707. doi:10.1038/s41586-023-06002-x

Liu, Z., Qian, Z., Wang, Y., and Wang, H. (2022). Necroptosis in pathogenesis of osteoarthritis and its therapeutic implications. *Zhejiang Da Xue Xue Bao Yi Xue Ban.* 51 (2), 261–265. doi:10.3724/zdxbyxb-2021-0402

Luo, J., Chen, S., Wang, L., Zhao, X., and Piao, C. (2022). Pharmacological effects of polydatin in the treatment of metabolic diseases: a review. *Phytomedicine* 102, 154161. doi:10.1016/j.phymed.2022.154161

Martinez, F. O., and Gordon, S. (2014). The M1 and M2 paradigm of macrophage activation: time for reassessment. *F1000Prime Rep.* 6, 13. doi:10.12703/P6-13

Moriwaki, S., Into, T., Suzuki, K., Miyauchi, M., Takata, T., Shibayama, K., et al. (2016). γ-Glutamyltranspeptidase is an endogenous activator of Toll-like receptor 4-mediated osteoclastogenesis. *Sci. Rep.* 6, 35930. doi:10.1038/srep35930

Mostafa, F., Abdel-Moneim, A., Abdul-Hamid, M., Galaly, S. R., and Mohamed, H. M. (2021). RETRACTED ARTICLE: polydatin and polydatin-loaded chitosan nanoparticles attenuate diabetic cardiomyopathy in rats. *J. Mol. Histol.* 52 (2), 135–152. doi:10.1007/s10735-020-09930-4

Napolitano, G., Fasciolo, G., and Venditti, P. (2021). Mitochondrial management of reactive oxygen species. *Antioxidants (Basel)* 10 (11), 1824. doi:10.3390/antiox10111824

Pan, X., Shan, H., Bai, J., Gao, T., Chen, B., Shen, Z., et al. (2022). Four-octyl itaconate improves osteoarthritis by enhancing autophagy in chondrocytes via PI3K/AKT/mTOR signalling pathway inhibition. *Commun. Biol.* 5 (1), 641. doi:10.1038/s42003-022-03592-6

Piao, L., Wu, D., Rui, C., Yang, Y., Liu, S., Liu, J., et al. (2023). The Bcr-Abl inhibitor DCC-2036 inhibits necroptosis and ameliorates osteoarthritis by targeting RIPK1 and RIPK3 kinases. *Biomed. Pharmacother.* 161, 114528. doi:10.1016/j.biopha.2023.114528

Rastogi, R. P., Singh, S. P., Hader, D. P., and Sinha, R. P. (2010). Detection of reactive oxygen species (ROS) by the oxidant-sensing probe 2',7'-dichlorodihydrofluorescein diacetate in the cyanobacterium Anabaena variabilis PCC 7937. *Biochem. Biophys. Res. Commun.* 397 (3), 603–607. doi:10.1016/j.bbrc.2010.06.006

Sang, X., Zhao, X., Yan, L., Jin, X., Wang, X., Wang, J., et al. (2022). Thermosensitive hydrogel loaded with primary chondrocyte-derived exosomes promotes cartilage repair by regulating macrophage polarization in osteoarthritis. *Tissue Eng. Regen. Med.* 19 (3), 629–642. doi:10.1007/s13770-022-00437-5

Shi, F., Ni, L., and Gao, Y. M. (2022). Tetrandrine attenuates cartilage degeneration, osteoclast proliferation, and macrophage transformation through inhibiting P65 phosphorylation in ovariectomy-induced osteoporosis. *Immunol. Invest* 51 (3), 465–479. doi:10.1080/08820139.2020.1837864

Sun, H., Sun, Z., Xu, X., Lv, Z., Li, J., Wu, R., et al. (2022). Blocking TRPV4 ameliorates osteoarthritis by inhibiting M1 macrophage polarization via the ROS/NLRP3 signaling pathway. *Antioxidants (Basel)* 11 (12), 2315. doi:10.3390/antiox11122315

Tang, D., Zhang, Q., Duan, H., Ye, X., Liu, J., Peng, W., et al. (2022). Polydatin: a critical promising natural agent for liver protection via antioxidative stress. *Oxid. Med. Cell Longev.* 2022, 1–14. doi:10.1155/2022/9218738

Wang, X., Wang, D., Deng, B., and Yan, L. (2023). Syringaresinol attenuates osteoarthritis via regulating the NF-κB pathway. *Int. Immunopharmacol.* 118, 109982. doi:10.1016/j.intimp.2023.109982

Wei, J., Liu, L., Li, Z., Lyu, T., Zhao, L., Xu, X., et al. (2022). Fire needling acupuncture suppresses cartilage damage by mediating macrophage polarization in mice with knee osteoarthritis. *J. Pain Res.* 15, 1071–1082. doi:10.2147/JPR.S360555

Yang, G., Wang, K., Song, H., Zhu, R., Ding, S., Yang, H., et al. (2022). Celastrol ameliorates osteoarthritis via regulating TLR2/NF-κB signaling pathway. *Front. Pharmacol.* 13, 963506. doi:10.3389/fphar.2022.963506

Ying, J., Wang, P., Shi, Z., Xu, J., Ge, Q., Sun, Q., et al. (2023). Inflammation-mediated aberrant glucose metabolism in subchondral bone induces osteoarthritis. *Stem Cells* 41 (5), 482–492. doi:10.1093/stmcls/sxad012

Yousef, A. I., Shawki, H. H., El-Shahawy, A. A., El-Twab, S. M. A., Abdel-Moneim, A., and Oishi, H. (2021). Polydatin mitigates pancreatic β-cell damage through its antioxidant activity. *Biomed. Pharmacother.* 133, 111027. doi:10.1016/j.biopha.2020.111027

Yuan, Y., Feng, G., Yang, J., Yang, C., and Tu, Y. H. (2022). Polydatin alleviates osteoporosis by enhancing the osteogenic differentiation of osteoblasts. *Eur. Rev. Med. Pharmacol. Sci.* 26 (12), 4392–4402. doi:10.26355/eurrev_202206_29078

Yue, L., and Berman, J. (2022). What is osteoarthritis? *JAMA* 327 (13), 1300. doi:10.1001/jama.2022.1980

Zhang, X., Fan, L., Wu, J., Xu, H., Leung, W. Y., Fu, K., et al. (2019). Macrophage p38α promotes nutritional steatohepatitis through M1 polarization. *J. Hepatol.* 71 (1), 163–174. doi:10.1016/j.jhep.2019.03.014

Zhao, X., Di, Q., Liu, H., Quan, J., Ling, J., Zhao, Z., et al. (2022). MEF2C promotes M1 macrophage polarization and Th1 responses. *Cell Mol. Immunol.* 19 (4), 540–553. doi:10.1038/s41423-022-00841-w

Zhou, L., Yu, P., Wang, T. T., Du, Y. W., Chen, Y., Li, Z., et al. (2022). Polydatin attenuates cisplatin-induced acute kidney injury by inhibiting ferroptosis. *Oxid. Med. Cell Longev.* 2022, 9947191–9947214. doi:10.1155/2022/9947191

Zhou, Y., Lin, S., Huang, Z., Zhang, C., Wang, H., Li, B., et al. (2023). Receptor-interacting protein 1 inhibition prevents mechanical stress-induced temporomandibular joint osteoarthritis by regulating apoptosis and later-stage necroptosis of chondrocytes. *Arch. Oral Biol.* 147, 105612. doi:10.1016/j.archoralbio.2022.105612

Q17
