# Supplementary material for: Polydatin retards the progression of osteoarthritis by maintaining bone metabolicbalance and inhibiting macrophage polarization
Source: Front Bioeng Biotechnol. 2025 Jan 7;12:1514483. doi: 10.3389/fbioe.2024.1514483 (PMC11747576; doi:10.3389/fbioe.2024.1514483)
Supplement: Supplementary file 3 [file Table2.DOCX]

| Gene | Forward Primer (5´-3´) | Reverse Primers (5´-3´) |
| --- | --- | --- |
| GAPDH | GGGGAGCCAAAAGGGTCATCATCT | GAGGGGCCATCCACAGTCTTCT |
| IL-6 | GGCGGATCGGATGTTGTGAT | GGACCCCAGACAATCGGTTG |
| TNF-α | GGAACACGTCGTGGGATAATG | GGCAGACTTTGGATGCTTCTT |
| iNos  Col II  Aggrecan  ADAMT4 | CTCTTCGACGACCCAGAAAAC  TGTTTGCAGAGCACTACTTGAA  GAAGTGGCGTCCAAACCAAC  CGTTCCGCTCCTGTAACACT | CAAGGCCATGAAGTGAGGCTT  ACCAGGGGAACCACTCTCAC  AGCTGGTAATTGCAGGGGAC  TTGAAGAGGTCGGTTCGGTG |

Table 1. Sequences of Primers Used for RT-PCR
