# Supplementary material for: Polydatin retards the progression of osteoarthritis by maintaining bone metabolicbalance and inhibiting macrophage polarization
Source: Front Bioeng Biotechnol. 2025 Jan 7;12:1514483. doi: 10.3389/fbioe.2024.1514483 (PMC11747576; doi:10.3389/fbioe.2024.1514483)
Supplement: Supplementary file 4 [file DataSheet1.PDF]

河北医科大学
HEBEI MEDICAL UNIVERSITY

Laboratory Animal Ethical and Welfare Committee
Hebei Medical University

实验动物福利伦理审查同意书

Approval of Laboratory Animal Ethical and Welfare

编号 Approval No.	IACUC-Hebmu-2024040

本《动物实验方案》经过河北医科大学实验动物福利伦理委员会审核，符合动物保护、动物福利和伦理原则，符合国家实验动物福利伦理的相关规定。

The protocol of animal experiment listed below was reviewed and approved by the Laboratory Animal Ethical and Welfare Committee of Hebei Medical University.

课题名称 Protocol Title	虎杖苷通过维持骨代谢平衡抑制巨噬细胞极化延缓骨关节炎的进展<br>Polydatin retards the progression of osteoarthritis by maintaining bone metabolic balance and inhibiting macrophage polarization				
申请人 Applicant	孙琪<br>Qi Sun	职称/学位 Title/Degree	博士研究生<br>PHD	邮箱 Email	59003946@hebmu.edu.cn
课题负责人 Principle Investigator (PI)	南欣雨<br>Xinyu Nan	职称/学位 Title/Degree	讲师<br>PHD	邮箱 Email	19101797@hebmu.edu.cn
院系(部门) Department	护理学院<br>School of nursing			申请日期 Application date	2024.6.05
动物种系 Species or Strains	C57BL/6			动物数量 Quantity	32
计划执行时间 Period of Protocol	2024.07-2025.07	实验动物使用许可证号 Number of Animal use permit	SYXK（冀）2020-002		
审查意见 Results of inspection	☑ 符合动物福利伦理要求，可以进行实验 Agree<br>☐ 调整方案后，可以进行实验 Agree after modify				
兽医师 Chief Veterinary Officer	[signature]			日期 Date	2024.6.12

河北医科大学实验动物福利伦理委员会

Laboratory Animal Ethical and Welfare Committee of Hebei Medical Univeristy

主任委员（或授权人）: [signature]

日　期:

地址: 河北　石家庄市　中山东路 361 号 河北医科大学 邮编：050017
Add: No. 361 East Road, Shijiazhuang, Hebei Province, P. R. China. 050017
