# Supplementary material for: Polydatin retards the progression of osteoarthritis by maintaining bone metabolicbalance and inhibiting macrophage polarization
Source: Front Bioeng Biotechnol. 2025 Jan 7;12:1514483. doi: 10.3389/fbioe.2024.1514483 (PMC11747576; doi:10.3389/fbioe.2024.1514483)
Supplement: Supplementary file 5 [file Presentation2.pdf]

Q5.1The First Hospital of Hebei Medical University, No.89 Donggang Road, 050000, Shijiazhuang, Hebei, China.

2Hebei Medical University, No. 361 Zhongshan East Road, 050000, Shijiazhuang, Hebei, China.

Q12. In line 1368, change "ZX" to "X-LZ".

Q16.10.4081/clr.2019.14780

Q17.page：1-11

Q19. Molecular docking between PD and NF-κB (Typical docking mode and binding site images.A:The protein residues are represented by the ribbon model. B:The space filling model intuitively reflects the spatial position of PD in the inhibitory binding pocket of NF-κB Q19 .C:Docking modes and binding sites.
