# Supplementary material for: Polydatin retards the progression of osteoarthritis by maintaining bone metabolicbalance and inhibiting macrophage polarization
Source: Front Bioeng Biotechnol. 2025 Jan 7;12:1514483. doi: 10.3389/fbioe.2024.1514483 (PMC11747576; doi:10.3389/fbioe.2024.1514483)
Supplement: Supplementary file 2 [file Table1.DOC]

Editor-in-Chief

October 19 2024

Dear Editor-in-Chief,

Thank you for your attention to the attached submission. I am enclosing a manuscript entitled “Polydatin retards the progression of osteoarthritis by maintaining bone metabolicbalance and inhibiting macrophage polarization” for publication consideration in the *Biomechanical and Biomaterial Advances in Degenerative Diseases of Bone and Joint*. Within this manuscript we found that **Polydatin (PD) significantly delays the progression of osteoarthritis induced by Anterior cruciate ligament transection (ACLT), effectively inhibits IL-1β-induced joint inflammation, bone metabolic remodeling and extracellular matrix degradation. In addition, paeoniflorin markedly suppresses the transmission of the NF-κB signaling pathway and reverses M1 polarization in macrophages induced by bacterial lipopolysaccharide (LPS). Taken together, PD might be a potential therapeutic agent for the prevention and treatment of osteoarthritis.** We are confident that our manuscript provide a clinical guidance for the treatment of intervertebral disc degeneration with diabetes mellitus and will be a valuable contribution to the journal in this field.

With the submission of this manuscript, I would like to undertake that this paper has not been published elsewhere and accepted for publication elsewhere, nor is it under editorial review with any other publications. I have formatted the manuscript to fit the style according to the requirements of *Biomechanical and Biomaterial Advances in Degenerative Diseases of Bone and Joint.* I hope this manuscript could be accepted and greatly improved in quality with you and reviewers’ great help.

Thanks for your attention and great help.

Sincerely,

**Chang-Cheng Liu, MD, PhD and Guo-bin Liu, MD, PhD**

Department of Orthopedic Surgery, The First Hospital of Hebei Medical University, No.89 Donggang Road, Yuhua District, Shijiazhuang, 050000, Hebei, China. E-mail: 57803022@hebmu.edu.cn/ liuguobin@hebmu.edu.cn
